# TH/TRs–*COL11A2* Axis Mediates Loss of a Differentiated Astrocyte State in Hypogyrified Brains

**DOI:** 10.1002/advs.77598

**Published:** 2026-09-16

**Authors:** Ying Zhang, Xiao Wang, Hongyan Ma, Pengcheng Lyu, Shuangjie Tian, Ruojun Zong, Jiani Chen, Gang Ren, Yongjiang Yang, Zichen Yang, Guosong Qin, Naishi Li, Yanfang Wang, Jianwei Jiao, Jianguo Zhao

**Affiliations:** ^1^ Key Laboratory of Organ Regeneration and Reconstruction Institute of Zoology Chinese Academy of Sciences Beijing China; ^2^ Beijing Institute for Stem Cell and Regenerative Medicine Beijing China; ^3^ State Key Laboratory of Animal Biotech Breeding Institute of Animal Science Chinese Academy of Agricultural Sciences (CAAS) Beijing China; ^4^ University of Chinese Academy of Sciences Beijing China; ^5^ Department of Animal Genetics Breeding and Reproduction College of Animal Science South China Agricultural University Guangzhou China; ^6^ Department of Endocrinology Key Laboratory of Endocrinology of National Health Commission Peking Union Medical College Hospital Chinese Academy of Medical Sciences and Peking Union Medical College Beijing China

**Keywords:** astrocyte differentiation, cerebral gyrification, COL11A2, congenital hypothyroidism, pig, thyroid hormone

## Abstract

Congenital hypothyroidism (CH) is a prevalent endocrine disorder associated with cerebral hypogyrification in neonates and young children, yet the vulnerable cell types and underlying molecular mechanisms remain elusive. Using a gyrencephalic CH pig model, we observed cerebral atrophy and cortical hypogyrification, mirroring key neuropathological features of CH patients. Histological analyses revealed that glial cells were more prominently affected than cortical neurons, with reduced differentiated astrocytic features in the cortex and subcortical white matter, together with oligodendrocyte‐lineage and myelination defects in the subcortical white matter. Single‐cell RNA sequencing showed that astrocytes displayed the most pronounced transcriptional response to CH, identifying them as a major TH‐responsive cell type under CH conditions. Notably, a COL11A2‐enriched differentiated astrocyte state, Astro‐2, was virtually absent in CH brains. COL11A2 was further validated as a target of the TH/TH‐receptors (TRs) axis, and its dysregulation impaired astrocyte differentiation and morphological maturation in mice, pigs, and humans. Our findings suggest an association between endocrine regulation of astrocyte development and cerebral gyrification, and uncover a COL11A2‐mediated mechanism by which TH deficiency disrupts astrocyte differentiation, providing potential insights into CH‐associated cortical malformations.

## Graphical abstract

Using a gyrencephalic congenital hypothyroidism pig model, this study reveals cerebral atrophy and cortical hypogyrification. Single‐cell sequencing identifies astrocytes as major TH‐responsive cells, with the COL11A2‐enriched Astro‐2 state nearly absent. COL11A2 is a TH/TR target, and its dysregulation impairs astrocyte differentiation across mice, pigs, and humans.

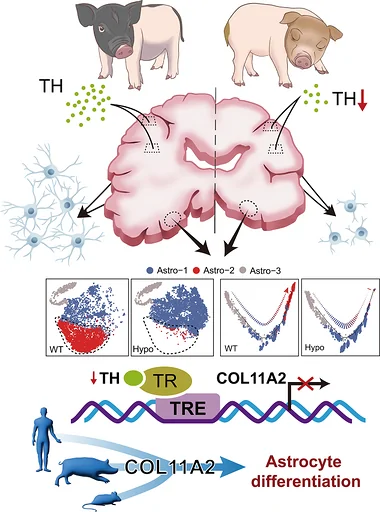

## 1 Introduction

Congenital hypothyroidism (CH), characterized by a deficiency of thyroid hormone (TH) during the perinatal period, results in severe developmental delays and intellectual impairment [1]. CH stands as one of the common causes of intellectual disability worldwide, with an estimated incidence ranging from 1 in 2000 to 1 in 4000 [2]. Given the critical role of TH in brain development, the developing fetus and neonate are particularly vulnerable to TH deficiency. Even with timely postnatal thyroxine (T4) treatment, irreversible brain damage may occur, profoundly impairing intelligence and motor function [3, 4, 5, 6]. Hence, a comprehensive understanding of the precise pathological features and mechanisms through which TH influences brain development will be instrumental in discovering enhanced treatment alternatives.

The cerebral cortex, constituting half of the volume of the human brain, boasts an intricately folded gyrencephalic structure essential for advanced cognitive capabilities. Malformations in its sulci and gyri can greatly disrupt brain function, leading to symptoms such as developmental delays and intellectual disabilities [7]. Patients with CH typically exhibit reduced sulci and gyri, as well as hypoplasia in the cortex and white matter. A clinical ultrasound examination of newborns diagnosed with CH revealed that 69% of these infants displayed delayed cerebral cortex development, along with diminished sulci and gyri [8]. In some cases, CH infants presented with bifrontal atrophy, a smoothing of the gyri in some regions, and hypomyelination in the white matter [9]. In severe instances, CH neonates and children showed lissencephaly, a condition marked by an unusually smooth cerebral surface [10, 11]. However, the vulnerable cell types and underlying molecular mechanisms responsible for cerebral hypogyrification in CH children remain unidentified.

In the brain, TH plays a crucial role in the development of both neurons and glia, influencing processes such as neurogenesis, gliogenesis, and synaptic plasticity. Glial cells, especially astrocytes, play a dual role in TH metabolism and signaling. They convert thyroxine (T4) into the biologically active triiodothyronine (T3) form through the predominant expression of type 2 deiodinase (DIO2) [12]. Moreover, astrocytes serve as direct targets of TH signaling through the expression of T3‐binding nuclear receptors (TRs), which are encoded by *THRA* and *THRB* [13]. These TRs act as ligand‐inducible transcription factors, regulating target gene expression in a hormone‐dependent manner [14].

Although the regulation of astrocyte development by TH has been reported, most studies are mainly restricted to in vitro systems or non‐cortical regions. In vitro studies showed that TH modulates astrocyte morphology, differentiation, and proliferation [15, 16]. In non‐neocortical brain tissues, hypothyroid rats exhibited reduced quantities of GFAP‐labeled astrocytes, decreased process branching, and delayed astrocyte differentiation in the cerebellum and hippocampus [13, 17, 18]. Similarly, *TRα* ^−/−^ mice displayed significant deficits in cerebellar astrocyte development and maturation [19]. However, relevant studies in cerebral tissue are rare. It remains unclear whether astrocyte abnormalities are associated with CH‐related cortical structural defects, and the mechanisms by which TH signaling regulates astrocyte development remain poorly understood.

Utilizing suitable animal models is crucial in understanding the pathological characteristics and molecular mechanisms of human diseases. Currently, systematic studies into the relationship between TH disruption and neurodevelopment predominantly center on rodent models [20]. However, in many cases, the rodent models only partially replicate the clinical symptoms of abnormal TH levels and typically do not exhibit significant neurological impairment [21, 22], unlike patients with severe neurodevelopmental deficits [23, 24]. Furthermore, rodents have a smooth‐surfaced cortex and are unable to mimic the abnormal cortical gyrification observed in CH patients. Pigs are ideal models for certain human diseases and have been extensively used in biomedical research [25, 26]. Their gyrencephalic brains share similarities with human brains in terms of size, anatomy, and neurobiological characteristics, including cerebral convolutions, the ratio of gray and white matter [27, 28], brain growth and development pattern [29, 30, 31], as well as the sequences and expression of astrocytic proteins [32, 33]. These parallels grant pigs an advantage over rodents in accurately simulating certain human brain diseases [34]. However, no systematic studies of thyroid dysfunction‐related neurodevelopment using pig models have been reported.

Our laboratory has previously generated a severe CH pig model with the *DUOX2^D409G/D409G^* mutation that replicates both genetic and typical pathological characteristics observed in humans, such as severe TH deficiency and anemia [26], providing a potential model for studying CH‐related neurodevelopmental abnormalities. Here, we observed that the CH pig model displayed phenotypic hallmarks of cerebral atrophy and cortical hypogyrification, similar to the clinical features of CH patients. Further analysis revealed that glial cells in both the cortex and subcortical white matter were more severely affected than cortical neurons in CH brains with hypogyrification. Applying single‐cell RNA sequencing (scRNA‐seq), we found that astrocytes exhibited pronounced transcriptional responses to CH, with several TH‐related effectors highly expressed and dysregulated in these cells. Remarkably, we identified a COL11A2‐enriched differentiated astrocyte state, which was nearly depleted in CH brains, consistent with impaired astrocyte differentiation. COL11A2 was validated as a target of the TH/TRs axis, and its dysregulation impaired astrocyte differentiation and morphological maturation across mice, pigs, and humans.

## 2 Results

### 2.1 Cerebral Atrophy and Cortical Hypogyrification in CH Pigs

**FIGURE 1:**
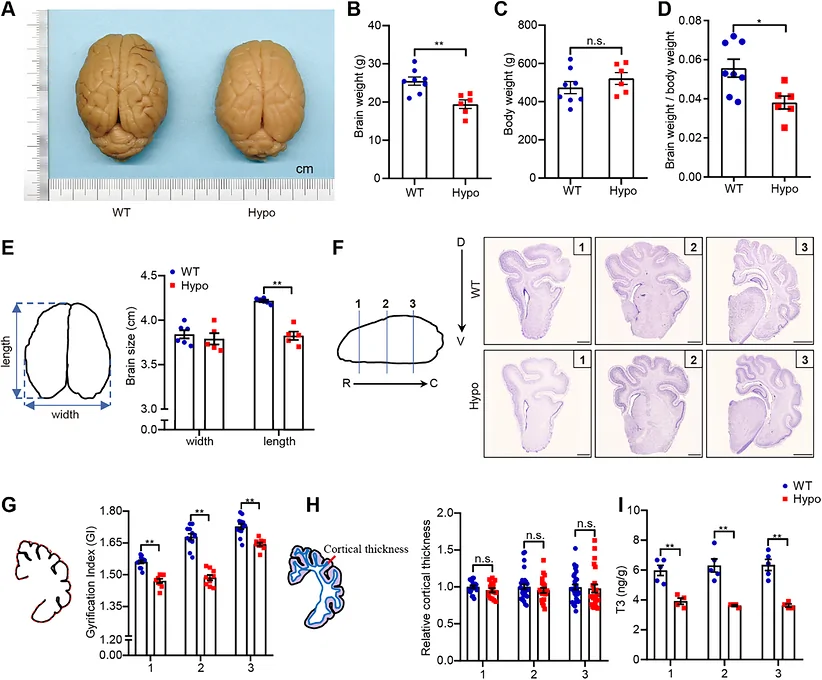
Characterization of cerebral dysplasia in Hypo pigs. (A) A photograph of the brains from a P0 WT (left) and a Hypo (right) pig. (B) Quantification of brain weight in WT and Hypo pigs (WT, n = 8 pigs; Hypo, n = 6 pigs). (C) Quantification of body weight in WT and Hypo pigs (WT, n = 8 pigs; Hypo, n = 6 pigs). (D) Quantification of brain weight normalized to body weight in WT and Hypo pigs (WT, n = 8 pigs; Hypo, n = 6 pigs). (E) The width and length of WT and Hypo brains (WT, n = 6 pigs; Hypo, n = 5 pigs). (F) Cresyl violet staining (Nissl staining) in coronal sections from the right hemispheres of WT and Hypo pigs at three regions along the rostrocaudal axis. R, rostral; C, caudal; D, dorsal; V, ventral. Scale bars, 2.5 mm (Regions 1 and 2); 5 mm (Region 3). (G) Gyrification index (GI) measurement between WT and Hypo brains at different regions in F (WT, n = 4 pigs; Hypo, n = 3 pigs, 3 slices per pig).GI was calculated as the ratio of the length of the complete contour (solid black line) to the outer contour (dotted red line). (H) The relative cortical thickness between WT and Hypo brains at different regions in F (WT, n = 4 pigs; Hypo, n = 3 pigs, 1 slice per pig, 5–10 locations per slice). (I) T3 (triiodothyronine) content per gram (g) of tissue in WT and Hypo brains at different regions in F (WT, n = 5 pigs; Hypo, n = 4 pigs). Data are shown as mean ± SEM. n.s., not significant; ^*^ *P* < 0.05; ^**^ *P* < 0.01; two‐tailed *t* test. WT: wild‐type, Hypo: hypothyroid.

To explore potential alterations in the brain structure of affected pigs and determine if these changes mimic the pathological characteristics observed in human clinical cases, we analyzed the postnatal day 0 (P0) brain phenotype in hypothyroid (hereafter referred to as Hypo) pigs. As shown in Figure 1A, overt cortical hypogyrification with reduced and shallow sulci was observed in the entire brain of the Hypo piglet compared to its wild‐type (WT) littermate. Quantitative analysis showed that body weight was not significantly different between WT and Hypo pigs, whereas brain weight was markedly reduced in Hypo pigs (Figure 1B,C; Table S1). Consistently, the brain weight‐to‐body weight ratio was significantly decreased in Hypo pigs, supporting a disproportionate reduction in brain growth under CH conditions (Figure 1D). Further measurement of gross brain size showed that brain length, but not brain width, was significantly reduced in Hypo pigs (Figure 1E), collectively suggesting the occurrence of cerebral atrophy.

To further investigate the differences between WT and Hypo brains, we performed coronal sections on the right hemispheres, targeting distinct regions for detailed analysis (Figure 1F, left). Cresyl violet staining in coronal sections further substantiated the widespread presence of hypogyrification throughout the entire Hypo brains (Figure 1F, right). For quantitative assessment of cortical folding, we calculated the gyrification index (GI) [35], revealing a significantly lower GI in Hypo brains compared to WT brains (Figure 1G). We next measured cortical thickness and found no significant differences between Hypo and WT brains across distinct regions (Figure 1H).

In addition, our previous study reported that serum T3, the active form of TH, was markedly decreased in Hypo pigs [26]. To ascertain alterations in T3 levels in the brains, we assessed the T3 content in Hypo and WT brains. As expected, T3 content was much lower in different regions of Hypo brains than in WT brains (Figure 1I), supporting an association between reduced local T3 levels and the abnormal brain phenotypes observed in Hypo pigs.

Taken together, CH piglets manifest cerebral atrophy and cortical hypogyrification, mirroring symptoms commonly observed in CH infants.

### 2.2 Alterations in Cortical Lamination With Preserved Neuronal Number, Migration‐Related Marker Expression, and Synaptic Marker Puncta in Hypo Brains

**FIGURE 2:**
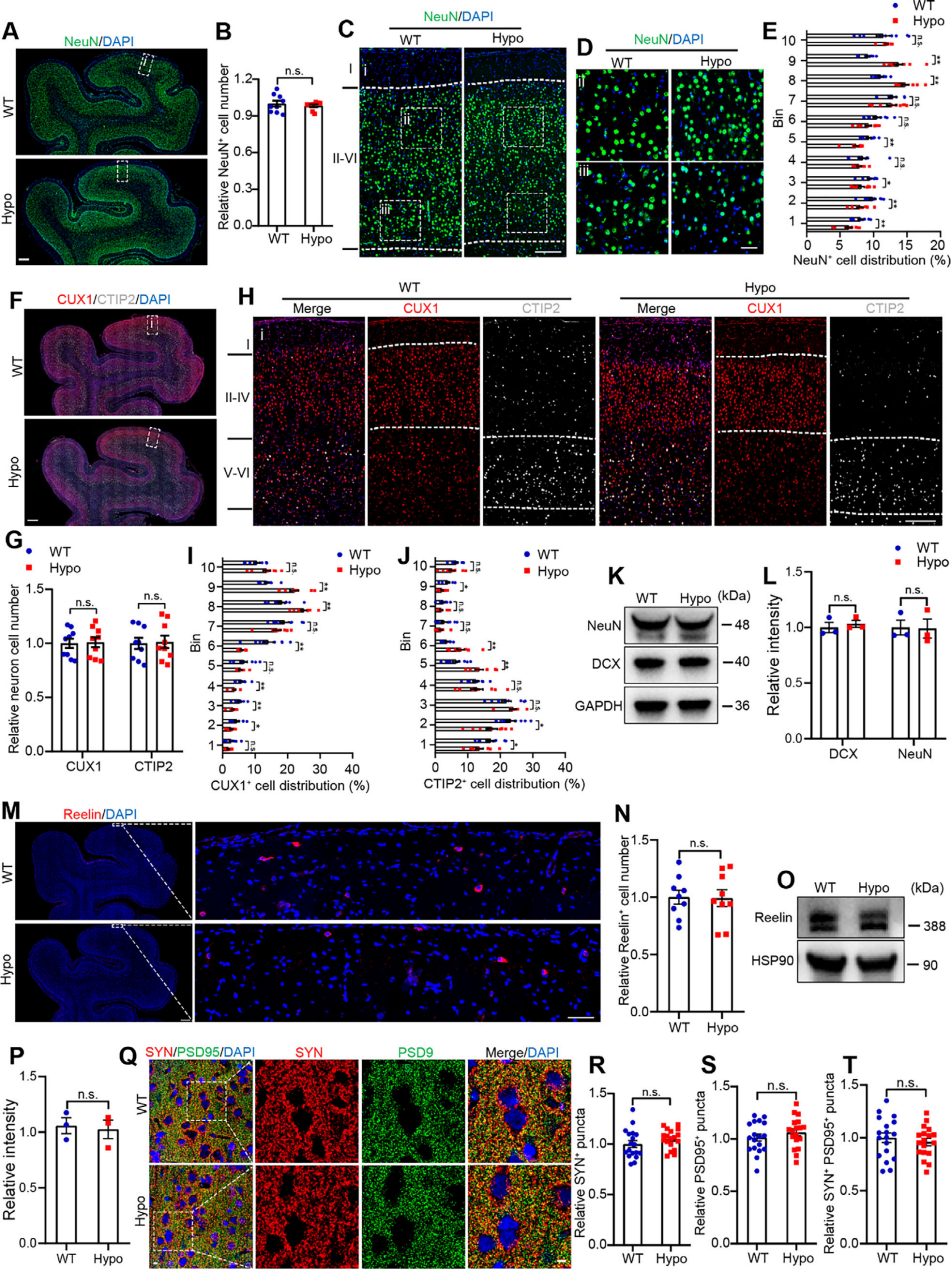
Analysis of cortical neurons in WT and Hypo pigs. (A) Immunofluorescence for NeuN, combined with DAPI staining, in the cortex of WT and Hypo pigs. Scale bar, 800 µm. (B) Quantification of NeuN^+^ neuron number throughout the cortex of A (WT, n = 3 pigs; Hypo, n = 3 pigs; 3 slices per pig). (C) Magnified views of the boxed areas in A. Scale bar, 200 µm. (D) Magnified views of the boxed areas in C. Scale bar, 50 µm. (E) Cortical bin analysis in C. The thickness of the CP, spanning from the outer border of layer II to the inner border of layer VI, was subdivided into 10 equal bins (from bin 10, superficial, to bin 1, deep) for NeuN^+^ neuron distribution quantification (WT, n = 3 pigs; Hypo, n = 3 pigs; 3 slices per pig). (F) Double immunofluorescence for CUX1 (upper‐layer neuronal marker) and CTIP2 (deep‐layer neuronal marker) from the cortex of WT and Hypo pigs at the first region along the rostrocaudal axis (Figure 1F). Scale bars, 800 µm. (G) Quantification of CUX1^+^ or CTIP2^+^ neuron number throughout the cortex of F (WT, n = 3 pigs; Hypo, n = 3 pigs; 3 slices per pig). (H) Magnified views of the boxed areas in F. Scale bar, 200 µm. (I–J) Cortical bin analysis in H for CUX1^+^ or CTIP2^+^ neuron distribution quantification (WT, n = 3 pigs; Hypo, n = 3 pigs; 3 slices per pig). (K) Western blot analysis of NeuN and DCX protein levels in WT and Hypo cortical tissues at the first region along the rostrocaudal axis shown in Figure 1F; GAPDH was used as loading control. (L) Quantification of NeuN and DCX protein levels (WT, n = 3 pigs; Hypo, n = 3 pigs). (M) Immunofluorescence for Reelin, combined with DAPI staining, in the cortex of WT and Hypo pigs. Scale bar, 50 µm. (N) Quantification of Reelin^+^ cell number (WT, n = 3 pigs; Hypo, n = 3 pigs; 3 slices per pig). (O) Western blot analysis of Reelin protein levels in WT and Hypo cortical tissues at the first region along the rostrocaudal axis shown in Figure 1F; HSP90 was used as loading control. (P) Quantification of Reelin protein levels (WT, n = 3 pigs; Hypo, n = 3 pigs). (Q) Immunofluorescence for SYN and PSD95, combined with DAPI staining, in cortical tissues of WT and Hypo pigs. The areas within the dashed boxes in the left panels are magnified and shown on the right. Scale bar, 5 µm. (R–T) Quantification of SYN^+^ puncta, PSD95^+^ puncta, and SYN^+^PSD95^+^ puncta in Q (WT, n = 3 pigs; Hypo, n = 3 pigs; 6 slices per pig). Data are shown as mean ± SEM. n.s., not significant; *P < 0.05; **P < 0.01; two‐tailed *t* test.

To determine whether cortical hypogyrification is associated with changes in cortical neurons, we performed histological and immunohistochemical analyses to examine neuronal lamination and distribution in Hypo pig brains. Both Nissl staining and NeuN immunostaining revealed that the total number of neurons remained comparable between Hypo and WT groups across different brain regions, although 10‐bin distribution analysis showed a relative enrichment of neurons in the upper cortical layers (bins 8–9), accompanied by a reduction in the middle (bin 5) and lower layers (bins 1–3) (Figure 2A–E; Figure S1A–H), indicating that the overall neuronal number is preserved despite altered cortical laminar organization.

To further assess the cortical laminar organization, we examined the expression of CUX1 and CTIP2, markers of upper‐layer (II‐IV, primarily corresponding to bins 6–10) and deep‐layer (V‐VI, primarily corresponding to bins 1–4) neurons, respectively (Figure 2F,H). The total number of CUX1^+^ and CTIP2^+^ neurons did not differ significantly between groups (Figure 2G, Figure S1G). Ten‐bin analysis, however, revealed layer‐specific alterations in Hypo pigs: CUX1^+^ neurons were enriched in the upper layers (bins 8–9) and reduced in the middle layers (bins 5 or 6), whereas CTIP2^+^ neurons decreased in the lower layers (bins 1 or 2) with a slight increase in the middle or lower layers (bins 5–6 or 3–4) (Figure 2I,J; Figure S1J). These results are consistent with the overall trends observed in Nissl and NeuN staining, further supporting that neuronal distribution is altered in a layer‐specific manner without changes in overall neuron number.

Given the preserved neuronal number but altered cortical laminar distribution, we next examined NeuN protein expression and the expression of migration‐related proteins, including DCX and Reelin. Western blot analysis showed that NeuN protein levels were comparable between WT and Hypo cortical tissues, consistent with the preserved NeuN^+^ neuron number observed by immunostaining (Figure 2K,L; Figure S1K,L). DCX, a microtubule‐associated protein expressed in immature and migrating neurons [36], also showed no significant difference between groups across distinct regions (Figure 2K,L; Figure S1K,L). Reelin, a large glycoprotein secreted predominantly by Cajal–Retzius cells in the marginal zone (layer I), is a key regulator of cortical development and neuronal positioning. Previous studies have shown that maternal hypothyroidism reduces Reelin expression, thereby impairing cortical lamination and neuronal migration [37]. However, in our CH model, the number of Reelin^+^ cells remained unchanged across distinct regions (Figure 2M,N; Figure S1M). Consistently, western blot analysis showed no significant change in Reelin protein levels across distinct regions (Figure 2O,P; Figure S1N,O). These results suggest that the laminar abnormalities observed here are unlikely to be primarily driven by broad alterations in DCX or Reelin expression.

In addition, we assessed synaptic organization in the cortex by immunostaining for synaptophysin (SYN), a presynaptic marker, and postsynaptic density protein‐95 (PSD95), a postsynaptic marker. To evaluate the structural organization of synaptic marker puncta, we quantified SYN^+^ puncta, PSD95^+^ puncta, and co‐localized SYN^+^PSD95^+^ puncta in the examined cortical regions. No significant differences were observed between WT and Hypo brains across distinct regions (Figure 2Q–T and Figure S1P), suggesting that the examined synaptic marker puncta are largely preserved in Hypo brains.

Collectively, these results suggest that although cortical laminar organization is altered in Hypo pig brains under CH conditions, the overall neuronal number, migration‐related marker expression, and examined synaptic marker puncta remain largely preserved.

### 2.3 Hypomyelination and Reduced Differentiated Astrocytic Features in Hypo Brains

**FIGURE 3:**
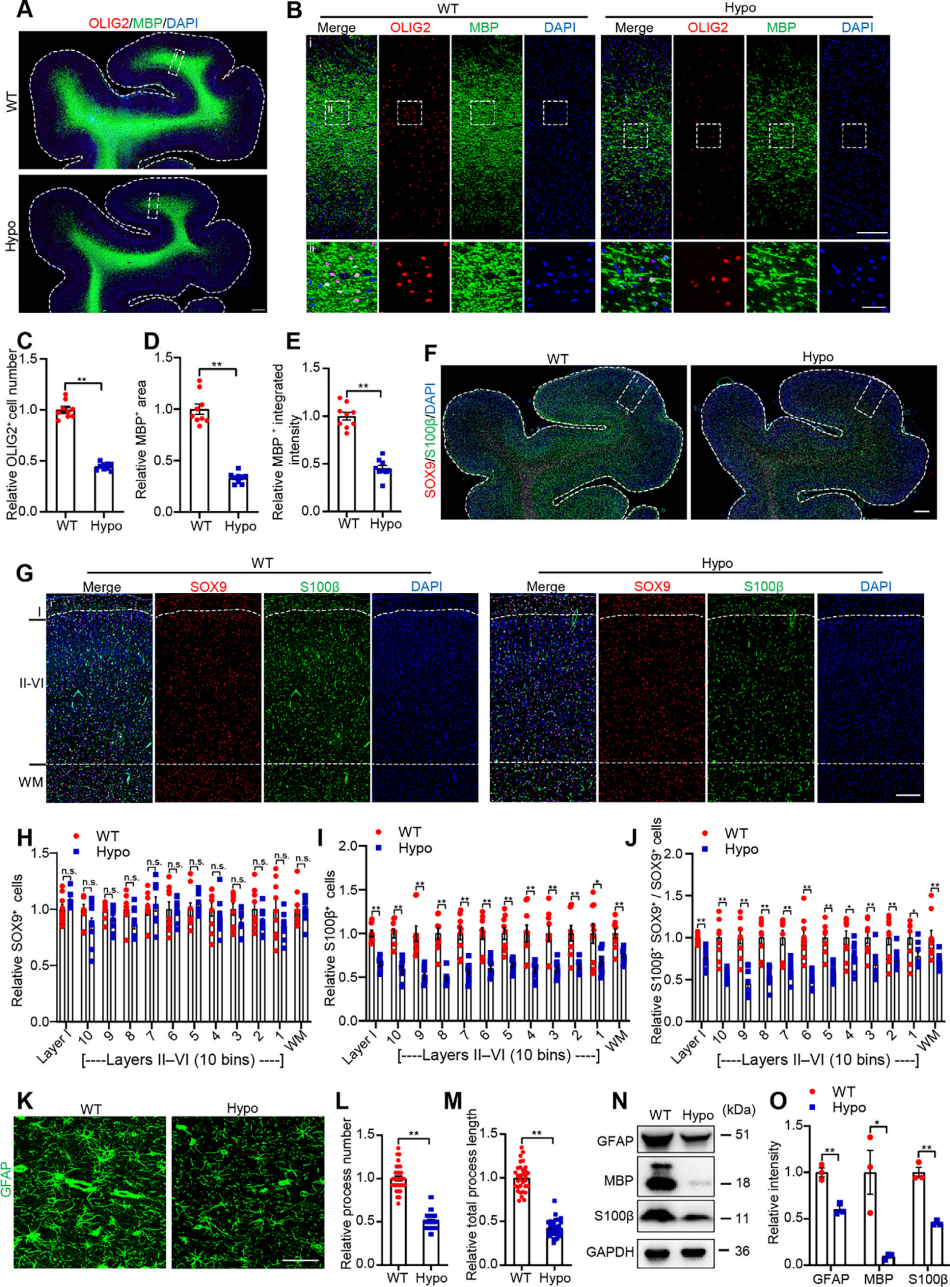
Characterization of glial dysplasia in Hypo cortex and subcortical white matter. (A) Double immunofluorescence for OLIG2 and MBP, combined with DAPI staining, in the subcortical white matter of WT and Hypo pigs at the first region along the rostrocaudal axis (Figure 1F). Scale bar, 800 µm. (B) Magnified views of the boxed areas in A. Scale bar, 200 µm. The areas within the boxes were magnified and are shown below. Scale bar, 50 µm. (C) Quantification of OLIG2^+^ cell number in A (WT, n = 3 pigs; Hypo, n = 3 pigs; 3 slices per pig). (D) Quantification of MBP^+^ area in A (WT, n = 3 pigs; Hypo, n = 3 pigs; 3 slices per pig). (E) Quantification of MBP^+^ integrated intensity in A (WT, n = 3 pigs; Hypo, n = 3 pigs; 3 slices per pig). (F) Double immunofluorescence for SOX9 and S100β, combined with DAPI staining, in the cortex and subcortical white matter of WT and Hypo pigs at the first region along the rostrocaudal axis shown in Figure 1F. Scale bar, 800 µm. (G) Magnified views of the boxed cortical and subcortical white matter regions in F. Scale bar, 200 µm. (H) Quantification of SOX9^+^ cell number across layer I, 10 equal bins spanning cortical layers II–VI, and white matter in G (WT, n = 3 pigs; Hypo, n = 3 pigs; 3 slices per pig). (I) Quantification of S100β^+^ cell number across layer I, 10 equal bins spanning cortical layers II–VI, and white matter in G (WT, n = 3 pigs; Hypo, n = 3 pigs; 3 slices per pig). (J) Quantification of S100β^+^SOX9^+^ cells among SOX9^+^ cells across layer I, 10 equal bins spanning cortical layers II–VI, and white matter in G (WT, n = 3 pigs; Hypo, n = 3 pigs; 3 slices per pig). (K) Representative images of GFAP^+^ astrocytes in the subcortical white matter of WT and Hypo pigs. Scale bar, 50 µm. (L) Quantification of GFAP^+^ process number in K (WT, n = 3 pigs; Hypo, n = 3 pigs; 30 cells per group). (M) Quantification of total GFAP^+^ process length in K (WT, n = 3 pigs; Hypo, n = 3 pigs; 30 cells per group). (N) Western blot analysis of GFAP, MBP, and S100β protein levels in tissues at the first region along the rostrocaudal axis shown in Figure 1F. (O) Quantification of GFAP, MBP, and S100β protein levels (WT, n = 3 pigs; Hypo, n = 3 pigs). Data are shown as mean ± SEM. n.s., not significant; *P < 0.05; **P < 0.01; two‐tailed *t* test.

To investigate the cellular alterations associated with cortical hypogyrification, we performed immunostaining to detect glial cells distributed in the cortex and subcortical white matter. We quantified oligodendrocyte‐lineage cells using OLIG2, a marker of the oligodendrocyte lineage, and observed a significant decrease in the number of OLIG2^+^ cells in the subcortical white matter of Hypo brains, compared with WT brains across distinct regions (Figure 3A–C; Figure S2A,B). Oligodendrocytes are responsible for producing the myelin sheath, and hypomyelination has been reported in CH patients presenting with cerebral hypogyrification [9]. Consistently, the area and integrated intensity of MBP‐positive signals, a marker of the myelin sheath, were noticeably reduced in Hypo brains (Figure 3A,B,D,E and Figure S2C,D).

In addition to oligodendrocytes, TH has profound effects on astrocyte number and morphology [18]. To assess astrocyte‐lineage and differentiated astrocytic populations, WT and Hypo brains were stained by double immunofluorescence for SOX9 and S100β. SOX9 was used as a nuclear marker of astrocyte‐lineage cells, whereas S100β was used to label differentiated astrocytic populations. Across layer I, 10 equal bins spanning cortical layers II‐VI, and white matter, the number of SOX9^+^ cells showed no significant difference between WT and Hypo brains (Figure 3F–H and Figure S2E,F), suggesting that the overall astrocyte‐lineage pool was largely preserved. In contrast, Hypo brains exhibited a marked reduction in S100β^+^ cells across cortical layers and white matter (Figure 3F,G,I and Figure S2G,H). Moreover, the proportion of S100β^+^SOX9^+^ cells among SOX9^+^ cells was decreased across distinct cortical layers and white matter regions (Figure 3J and Figure S2I,J). These findings indicate a reduction in S100β^+^ differentiated astrocytic features, rather than a broad loss of the overall SOX9^+^ astrocyte‐lineage pool.

To further evaluate astrocyte morphology, we analyzed GFAP^+^ astrocytes in the subcortical white matter, where GFAP expression is primarily detected [38]. GFAP^+^ astrocytes in Hypo brains displayed marked morphological abnormalities, including fewer processes and reduced total process length (Figure 3K–M and Figure S2K,L). Western blot analysis further supported these immunostaining results, showing decreased protein levels of GFAP, MBP, and S100β in Hypo tissues compared with WT controls across distinct regions (Figure 3N,O and Figure S2M–P).

To determine whether altered glial proliferation contributed to these phenotypes, we performed SOX9 and Ki67 co‐staining to assess proliferating astrocyte‐lineage cells in the VZ/SVZ, cortex, and white matter. Quantification of SOX9^+^Ki67^+^ cells among SOX9^+^ cells showed no significant differences between WT and Hypo brains in the VZ/SVZ across distinct regions (Figure S2Q,R). Similarly, the proportion of SOX9^+^Ki67^+^ cells among SOX9^+^ cells was not significantly altered across layer I, 10 equal bins spanning cortical layers II‐VI, and white matter across distinct regions (Figure S2S–V). These results suggest that the reduction in S100β^+^ and GFAP‐associated astrocytic features is not primarily attributable to altered proliferation of SOX9^+^ astrocyte‐lineage cells at this developmental stage.

Collectively, these results indicate that CH exerts a more pronounced impact on glial development and myelination in both the cortex and subcortical white matter than on cortical neurons in Hypo pigs. These glial alterations may be associated with the aberrant hypogyrification observed in Hypo brains.

### 2.4 Single‐Cell Transcriptome Profiling Identifies Astrocytes as Major TH‐Responsive Cells Under CH

**FIGURE 4:**
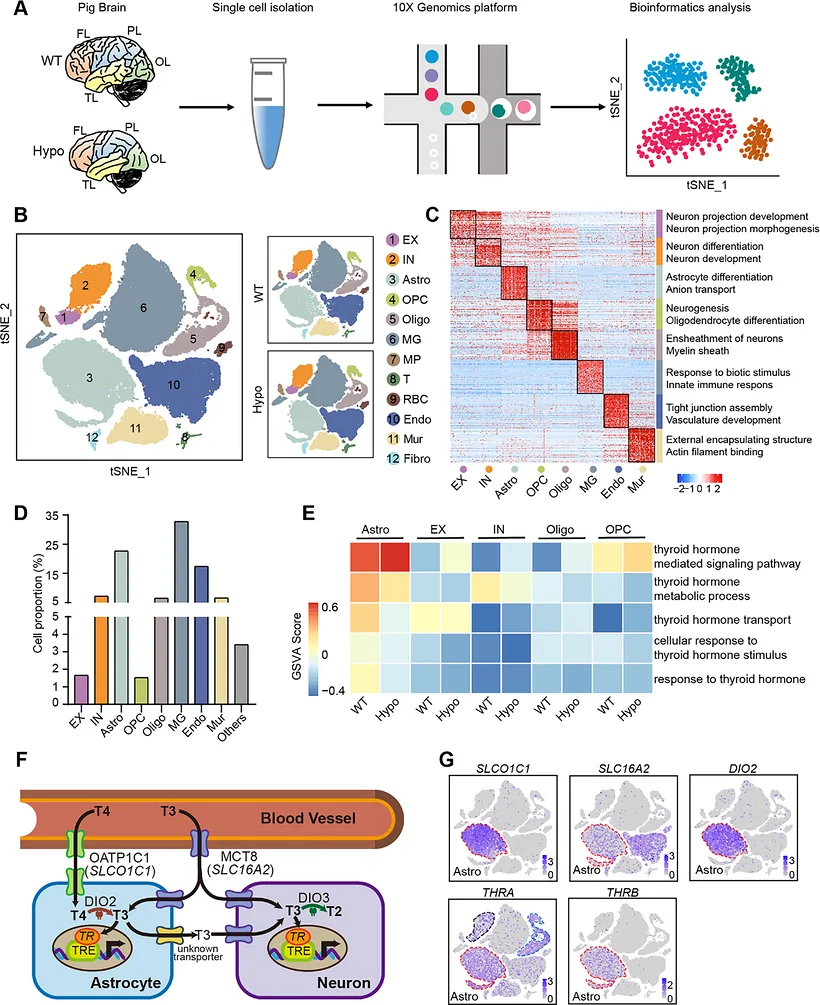
Cell type identification in pig brains by single‐cell RNA sequencing (scRNA‐seq). (A) Flowchart overview of scRNA‐seq of different brain regions, including the frontal lobe (FL), parietal lobe (PL), occipital lobe (OL), and temporal lobe (TL), from a WT and a Hypo pig. (B) Visualization of the scRNA‐seq data using a *t*‐SNE plot. The combined dataset is shown on the left, and the corresponding WT and Hypo distributions are shown on the right. EX, excitatory neurons; IN, inhibitory neurons; Astro, astrocytes; MG, microglia; Oligo, oligodendrocytes; OPC, oligodendrocyte progenitor cells; Endo, endothelial cells; Mur, mural cells; MP, macrophages; RBC, red blood cells; T, T cells; Fibro, fibroblasts. (C) Heatmap displaying the expression profiles of the top 100 specifically expressed genes in different cell types, accompanied by their enriched functional annotations on the right. (D) Bar plot showing the proportion of major cell types. (E) Heatmap showing TH metabolism and action‐related pathway activities scored per cell type by GSVA between WT and Hypo conditions. (F) Schematic model of the functional roles of TH effector genes in the brain. (G) Distribution of individual TH effector gene expression projected onto the *t*‐SNE plot. The red, blue, and black dashed boxes indicate astrocytes, oligodendrocyte lineage cells, and neurons, respectively.

To systematically identify the cellular targets and dysregulated molecular pathways underlying the observed changes in Hypo brains, we applied whole‐cell dissociation followed by scRNA‐seq analysis of the cerebral cortex and its adjoining subcortical white matter from the four lobes of WT and Hypo brains (Figure 4A). After stringent cell filtration, the high‐quality transcriptomes of 76419 (WT: 39,042; Hypo: 37377) single cells were retained for subsequent analyses (Figure S3A–C). Unsupervised clustering yielded 23 cell clusters, which were subsequently annotated into 12 major cell types according to their individual gene‐expression signatures and previously reported cell‐type markers [39, 40, 41, 42, 43] (Figure 4B and Figure S3D,E). These included excitatory neurons (EX, *NEUROD2* ^+^ and *TBR1* ^+^), inhibitory neurons (IN, *DLX1* ^+^ and *GAD2* ^+^), astrocytes (Astro, *AQP4* ^+^ and *GFAP* ^+^), microglia (MG, *AIF1* ^+^ and *CX3CR1* ^+^), oligodendrocytes (Oligo, *MBP* ^+^ and *PLP1* ^+^), oligodendrocyte progenitor cells (OPC, *GPR17^+^* and *TNR* ^+^), endothelial cells (Endo, *FLT1* ^+^ and *FN1* ^+^), mural cells (Mur, *RGS5^+^* and *HIGD1B* ^+^), macrophages (MP, *CD163^+^*), T cells (T, *CXCR4^+^*), red blood cells (RBC, *HBB* ^+^) and fibroblasts (Fibro, *COL1A1* ^+^) (Figure S3F). Gene Ontology (GO) analysis of the top 100 marker genes for main cell types confirmed unique functional characteristics corresponding to their cell identities, further confirming the accuracy of cell type identification (Figure 4C). Cell‐type proportion analysis revealed a relative enrichment of glial cells over neurons (Figure 4D), likely attributable to the preferential loss of neurons caused by their susceptibility to whole‐cell dissociation [44].

To assess cell‐type‐specific effects of CH, we performed gene set variation analysis (GSVA) to evaluate TH metabolism and action‐related pathway activity across distinct cell types. Astrocytes consistently exhibited higher GSVA scores across all TH‐related pathways compared to excitatory neurons, inhibitory neurons, and oligodendrocyte lineage cells (Figure S3G). Moreover, these pathways were markedly altered in astrocytes between WT and Hypo conditions (Figure 4E), indicating that astrocytes exhibit prominent TH‐related pathway activity and pronounced transcriptional alterations under CH conditions relative to other cell types. Consistent with this observation, astrocytes exhibited 394 differentially expressed genes (DEGs), whereas OPCs and oligodendrocytes exhibited 161 and 174 DEGs, respectively, and excitatory neurons showed only 68 DEGs, of which approximately one‐third remained unannotated (Figure S3H–K). These findings suggest that glial cells, particularly astrocytes, may exhibit a more robust transcriptional response to CH than neurons.

The observed alterations in TH metabolism and action‐related pathway activity were largely driven by variations in the expression of core TH effectors [45], including transporters (*SLCO1C1* and *SLC16A2*), deiodinase (*DIO2*), and receptors (*THRA* and *THRB*), which prompted further investigation of their cell‐type‐specific expression (Figure 4F). An intriguing pattern of co‐expression among these genes was observed, primarily within astrocytes, where their expression levels were relatively high (Figure 4G). Of note, *THRA* was expressed not only in neurons and the oligodendrocyte lineage, but also at relatively high expression levels in astrocytes (Figure 4G). Importantly, the co‐expression of both *THRA* and *THRB* in astrocytes highlights their role as direct target cells of TH (Figure 4G).

Altogether, these findings demonstrate that several TH effectors are highly expressed in astrocytes and that astrocytes exhibit pronounced transcriptional responses to Hypo conditions at single‐cell resolution, further supporting astrocytes as major TH‐responsive cells under CH conditions.

### 2.5 Marked Depletion of a COL11A2‐Enriched Differentiated Astrocyte State in Hypo Brains

**FIGURE 5:**
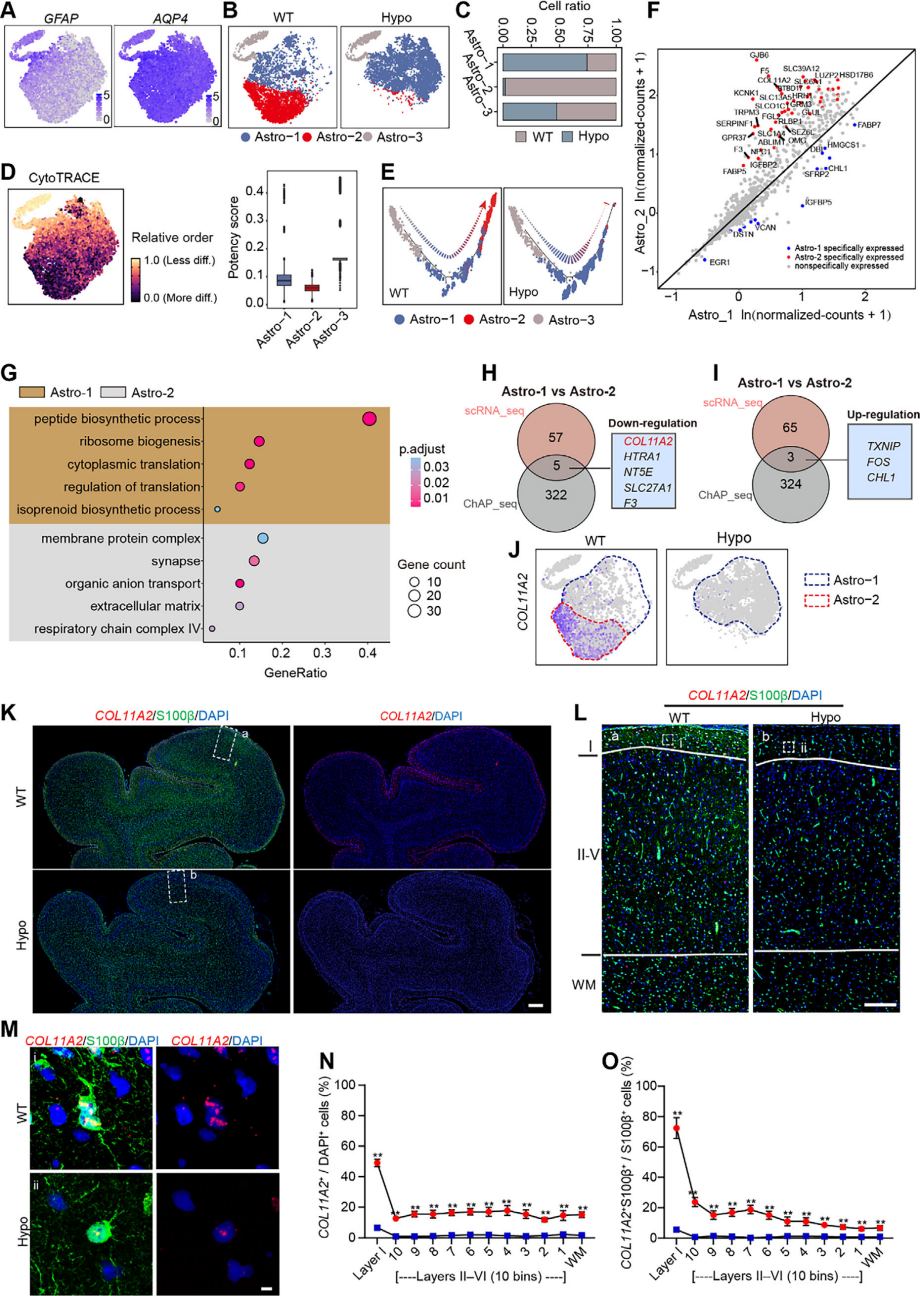
Changes in the transcriptional profiles of astrocyte subpopulations in Hypo brains. (A) Expression levels of typical astrocyte marker genes visualized on *t*‐SNE plots. (B) Distributions of astrocyte subpopulations in WT and Hypo brains visualized on *t*‐SNE plots. Cells are colored by subtype. Astro‐1, Astrocytes‐1; Astro‐2, Astrocytes‐2; Astro‐3, Astrocytes‐3. (C) Cell ratios of WT and Hypo astrocytes in different subtypes. (D) Left, CytoTRACE analysis depicting the differentiation states of astrocyte subpopulations; Right, developmental potency score of each subpopulation. (E) Pseudotime trajectory of WT and Hypo astrocyte subpopulations. (F) Scatter plot showing the specifically expressed genes in Astro‐1 and Astro‐2. (G) Representative GO terms of specifically expressed genes in Astro‐1 and Astro‐2, respectively. (H) Left, Venn plot showing the number of overlapping genes between downregulated DEGs of Astro‐1 compared with Astro‐2 and ChAP‐seq by Chatonnet et al. The overlapping genes are listed on the right. (I) Left, Venn plot showing the number of overlapping genes between upregulated DEGs of Astro‐1 compared with Astro‐2 and ChAP‐seq by Chatonnet et al. The overlapping genes are listed on the right. (J) Expression levels of *COL11A2* in the subpopulation of WT and Hypo astrocytes visualized on *t*‐SNE plots. (K) RNA fluorescence in situ hybridization for *COL11A2* combined with S100β immunofluorescence and DAPI staining in WT and Hypo pig brains. Scale bar, 800 µm. (L) Magnified views of the boxed cortical areas in K. Layer I, cortical layers II–VI, and white matter (WM) are indicated. Scale bar, 200 µm. (M) Magnified views of the boxed areas in L. Scale bar, 5 µm. (N) Quantification of the proportion of*COL11A2* ^+^ cells among DAPI^+^ cells across layer I, 10 equal bins spanning cortical layers II–VI, and white matter in L (WT, n = 3 pigs; Hypo, n = 3 pigs; 3 slices per pig). (O) Quantification of the proportion of*COL11A2* ^+^S100β^+^ cells among S100β^+^ cells across layer I, 10 equal bins spanning cortical layers II–VI, and white matter in L (WT, n = 3 pigs; Hypo, n = 3 pigs; 3 slices per pig). Data are shown as mean ± SEM, ^**^ *P* < 0.01 (two‐tailed *t* test).

We further investigated the subpopulation heterogeneity of astrocytes in different regions of WT and Hypo pigs. Based on astrocyte gene‐expression signatures, we identified three transcriptomically unique subpopulations, hereafter referred to as Astro‐1, Astro‐2 and Astro‐3 (Figure 5A,B; Figure S4A). The overall astrocyte proportion showed no obvious difference between WT and Hypo brains, either in the combined dataset or across the examined brain regions (Figure S4B). However, the astrocyte‐state composition appeared altered under Hypo conditions. The relative proportion of Astro‐3 remained stable between WT and Hypo brains, whereas Astro‐1 was increased and Astro‐2 was nearly absent in Hypo samples (Figure 5B,C). Importantly, these trends were consistently observed across different brain regions (Figure S4C,D).

To assess the developmental status of each subtype, we performed CytoTRACE analysis, which revealed that Astro‐3 exhibited the least differentiated state, followed by Astro‐1, and Astro‐2 as the most differentiated (Figure 5D). Pseudotime analysis further revealed a progressive trajectory from Astro‐3 to Astro‐1 and finally to Astro‐2 (Figure 5E). The marked loss of Astro‐2, alongside the accumulation of intermediate‐state Astro‐1 cells, suggests an altered distribution of astrocyte states along the differentiation trajectory under CH conditions. Notably, Astro‐2 expresses the canonical gray matter astrocyte marker *GJB6* [46], suggesting a cortical‐associated differentiated astrocyte state.

Given the near depletion of Astro‐2 under CH conditions, we focused on WT samples to identify subtype‐specific genes (Figure 5F), followed by GO enrichment analysis for Astro‐1 and Astro‐2 (Figure 5G). Astro‐1‐specific genes were predominantly enriched in pathways related to protein synthesis, including peptide biosynthetic process, cytoplasmic translation, ribosome biogenesis, and regulation of translation (Figure 5G and Table S2). Additionally, significant enrichment was observed in the isoprenoid biosynthetic process (Figure 5G and Table S2), suggesting a potential involvement of Astro‐1 in lipid metabolism and membrane‐associated signaling. By contrast, Astro‐2 signature genes were predominantly involved in pathways related to organic anion transport, synapse function, extracellular matrix organization, respiratory chain complex IV, and membrane protein complexes (Figure 5G and Table S2), suggesting distinct roles in astrocyte function.

To identify potential TH‐regulated genes that may contribute to the subtype alteration observed under CH conditions, we compared our scRNA‐seq data with the ChAP‐seq data reported by Chatonnet et al., who analyzed genome‐wide T3 target genes in neural progenitor cells by overexpressing TRs [47]. Specifically, we intersected the DEGs identified between Astro‐1 and Astro‐2 in WT samples with the reported T3 target genes from the ChAP‐seq dataset. This analysis identified eight overlapping genes, including five downregulated genes (*COL11A2*, *HTRA1*, *NT5E*, *SLC27A1* and *F3*; Figure 5H) and three upregulated genes (TXNIP, FOS, and CHL1; Figure 5I). Notably, among these genes, only *COL11A2* exhibits preferential enrichment in the Astro‐2 state (Figure 5J and Figure S4E), suggesting that *COL11A2* may represent a state‐specific molecular signature of differentiated Astro‐2 cells and may be linked to the selective loss of this state under TH deficiency.

To further validate the spatial distribution of the Astro‐2‐associated gene *COL11A2*, we performed RNA fluorescence in situ hybridization (FISH) for *COL11A2* combined with S100β immunofluorescence in WT and Hypo pig brains (Figure 5K–M). *COL11A2* RNA FISH analysis revealed that *COL11A2* ^+^ cells exhibited a layer‐biased distribution pattern in WT brains, with the highest proportion observed in layer I. *COL11A2* ^+^ cells were also detected across cortical layers II‐VI and white matter (Figure 5N). Combined *COL11A2* RNA FISH and S100β immunofluorescence further detected *COL11A2* RNA signals in a subset of S100β^+^ cells in WT brains, with the highest *COL11A2* ^+^S100β^+^/S100β^+^ proportion observed in layer I (Figure 5O). By contrast, in Hypo brains, *COL11A2* RNA signals were markedly reduced across the examined cortical and white matter regions, with COL11A2^+^ cells reduced to near‐background levels (Figure 5K–O). In addition, *COL11A2* RNA signals were detected in S100β^+^SOX9^+^ cells (Figure S4F).

These results are consistent with the scRNA‐seq finding that *COL11A2* is preferentially enriched in the Astro‐2 state and support its association with a differentiated astrocyte state. Moreover, the reduction of *COL11A2* ^+^S100β^+^ cells, in the context of preserved SOX9^+^ astrocyte‐lineage abundance, provides spatial and molecular support for the reduction of a *COL11A2*‐associated differentiated astrocyte state in both cortical and white matter regions of Hypo brains (Figure 3G–J).

Additionally, given the significant reduction in oligodendrocyte‐lineage cells and MBP^+^ myelin signals in the subcortical white matter of Hypo pigs (Figure 3A–E), we analyzed the regional heterogeneity of oligodendrocyte lineage cells in WT and Hypo brains. We identified one OPC subpopulation and three oligodendrocyte subpopulations (Oligo‐1‐3) (Figure S5A,B). Pseudotime trajectory analysis revealed a maturation path from OPCs through Oligo‐3 and Oligo‐2 to the more differentiated Oligo‐1 state (Figure S5C). The relative proportions of these oligodendrocyte‐lineage states showed a trend toward increased OPCs and reduced Oligo‐1 cells in the Hypo group compared with WT, a pattern observed across all four examined brain regions, whereas the proportions of Oligo‐2 and Oligo‐3 exhibited only slight regional variations (Figure S5D–F). In line with the increased OPC proportion, PDGFRα/OLIG2 immunofluorescence showed an increased fraction of PDGFRα^+^OLIG2^+^ OPC cells within the OLIG2^+^ lineage in Hypo subcortical white matter (Figure S5G,H). Together with the reduced Oligo‐1 abundance and decreased MBP signals, these findings suggest impaired progression from OPCs toward differentiated, myelinating oligodendrocytes under Hypo conditions. We further found that *COL11A2* was also weakly expressed in OPCs and Oligo‐3 (Figure S4E), and its expression was reduced in both cell types in the Hypo group compared to WT (Figure S5I).

Collectively, these findings reinforce the influence of CH on both astrocytes and the oligodendrocyte lineage at the single‐cell level, with astrocytes exhibiting greater transcriptional responsiveness. These observations suggest that *COL11A2* represents a state‐associated molecular signature preferentially enriched in the differentiated Astro‐2 state with weaker expression in oligodendrocyte‐lineage cells, and may act as a candidate TH‐responsive gene involved in astrocyte development.

### 2.6 TH/TRs Axis Promotes Astrocyte Differentiation by Regulating COL11A2

**FIGURE 6:**
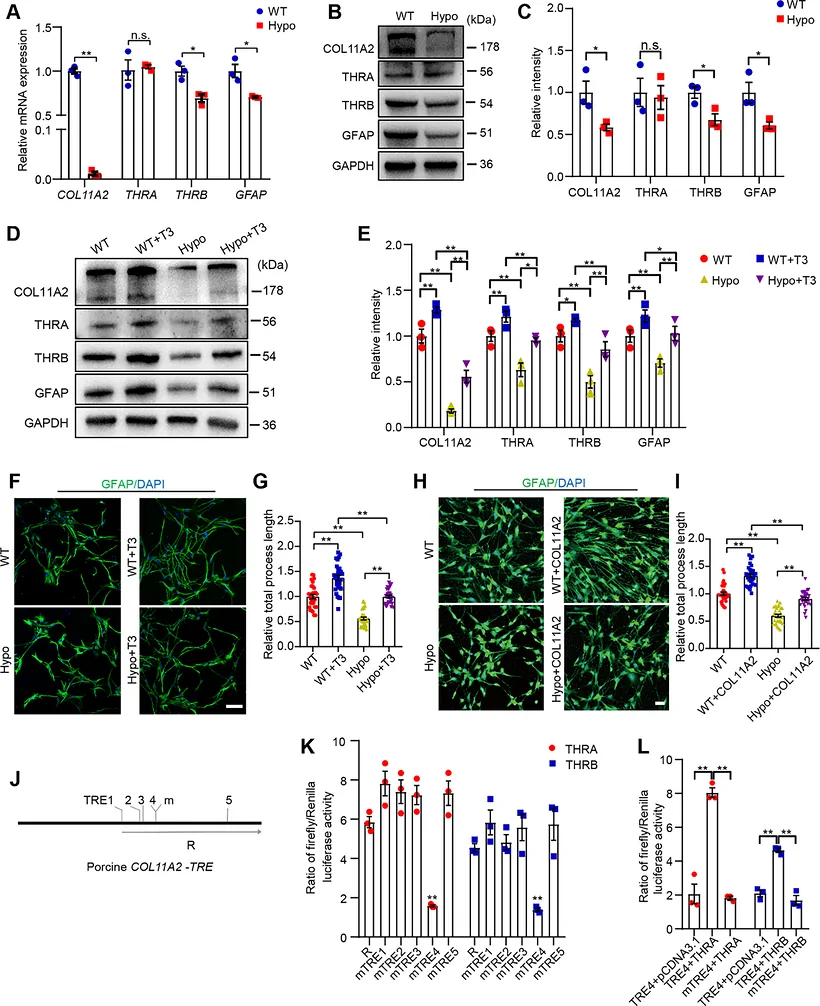
TH/TRs axis promotes astrocyte differentiation by regulating COL11A2. (A) qRT‐PCR analysis of the expression levels of *COL11A2*, *THRA*, *THRB*, and *GFAP* in the cortex from WT and Hypo pigs (WT, n = 3 pigs; Hypo, n = 3 pigs). (B) Western blot analysis of the expression levels of COL11A2, THRA, THRB, and GFAP in the cortex from WT and Hypo pigs. (C) Quantification of the protein levels in B (WT, n = 3 pigs; Hypo, n = 3 pigs). (D) Western blot analysis of the expression levels of COL11A2, THRA, THRB and GFAP in WT and Hypo astrocytes, differentiated from porcine neural stem cells, with or without T3 treatment. (E) Quantification of the protein levels in D (n = 3 biological replications). (F) Immunofluorescence for GFAP, combined with DAPI staining, in WT and Hypo astrocytes with or without T3 treatment. Scale bars, 200 µm. (G) Quantification of total GFAP^+^ process length in (F) (n = 3 biological replications, 30 cells per group). (H) Immunofluorescence for GFAP, combined with DAPI staining, in WT and Hypo astrocytes with or without 200 ng/mL COL11A2 treatment. Scale bars, 50 µm. (I) Quantification of total GFAP^+^ process length in H (n = 3 biological replications, 30 cells per group). (J) Prediction of putative TREs located in the porcine *COL11A2* promoter (R: region, m: mutation). (K) Luciferase assay of the reporters containing the mutated TRE1‐5 of the truncated *COL11A2* promoter co‐transfected with *THRA* or *THRB* in HEK293T cells (n = 3 biological replications). (L) Luciferase assay of TRE4 and the mutated TRE4 in response to any TR isoform (n = 3 biological replications). Data are shown as mean ± SEM. n.s., not significant; *P < 0.05; **P < 0.01 (two‐tailed t test or one‐way ANOVA followed by Holm–Sidak's multiple comparisons test).

To validate whether COL11A2 is regulated by the TH/TRs axis, we initially examined its cortical expression in WT and Hypo pig brains via western blot and qRT‐PCR analyses. COL11A2 was significantly decreased at both mRNA and protein levels in Hypo brains compared to WT (Figure 6A–C). This expression pattern aligned with that of THRB and GFAP (Figure 6A–C), suggesting that COL11A2 expression may be positively regulated by THRB in brain tissues. We further investigated the expression pattern of COL11A2 in vitro by isolating primary cells from the prefrontal cortex of newborn piglets. Immunostaining showed that COL11A2 was expressed in SOX2‐labeled neural stem cells (NSCs), BLBP‐labeled astrocyte precursor cells, and S100β‐labeled astrocytes (Figure S6A). Importantly, COL11A2 showed positive signals in cells labeled with THRA and THRB (Figure S6A). Subsequently, we isolated porcine NSCs (pNSCs) and cultured them in differentiation medium. pNSCs, co‐labeled with BLBP and SOX2, primarily differentiated into GFAP^+^ astrocytes after 7 days of culture (Figure S6B). To evaluate the inducibility of TH on *COL11A2* expression, we treated pNSC‐derived astrocytes with T3. qRT‐PCR analysis revealed a dose‐ and time‐dependent upregulation of *COL11A2* upon T3 treatment (Figure S6C,D). Concurrent upregulation of both *THRA* and *THRB* was also observed (Figure S6C,D). These findings suggest that *COL11A2* responds to T3 stimulation and is potentially regulated by TRs in astrocytes.

To further assess the role of COL11A2 under TH deficiency, we compared pNSC‐derived astrocytes from WT and Hypo newborn piglets, treated with or without 100 nM T3 for 24 h. Western blot analysis revealed a significant reduction in COL11A2, THRA, THRB, and GFAP expression in Hypo astrocytes compared to WT (Figure 6D,E). T3 treatment significantly upregulated these genes in WT astrocytes and partially restored their expression in Hypo astrocytes (Figure 6D,E). Since T3 influences the morphological differentiation of astrocytes [13, 17, 18], we proceeded to examine the morphological alterations in Hypo‐derived astrocytes. Immunostaining showed that morphological differentiation of Hypo GFAP^+^ astrocytes was hindered, as evidenced by reduced total process length (Figure 6F,G). Notably, T3 treatment increased process length in both WT and Hypo GFAP^+^ astrocytes (Figure 6F,G), aligning with the observed protein expression patterns (Figure 6D,E). These observations suggest a potential link between T3/TR‐mediated COL11A2 regulation and astrocyte morphological differentiation.

Given that COL11A2 is an extracellular matrix protein [48], we detected its amount in the supernatant of astrocyte culture medium after 24‐h T3 treatment using ELISA. The results confirmed COL11A2 secretion by astrocytes and its T3‐dependent regulation (Figure S6E). Furthermore, immunofluorescence analysis demonstrated that treatment with exogenous COL11A2 for 24–48 h significantly enhanced astrocytic process elongation in both WT and Hypo groups, partially rescuing the morphological deficits caused by the Hypo condition (Figure 6H,I). These results suggest that COL11A2 may regulate astrocyte differentiation in an autocrine manner.

TRs regulate their target genes via the binding of specific TH response elements (TREs) in the promoter region [49]. Bioinformatics analysis indicated that there are five putative TREs in the region (R) of the porcine *COL11A2* promoter sequence (Figure 6J; Table S3). To test whether these predicted TREs mediate TR‐dependent transcriptional activity of the porcine *COL11A2* promoter in vitro, plasmids containing region R and porcine TR isoforms were co‐transfected into HEK293T cells. After 24 to 48 h, a dual‐luciferase reporter assay revealed that only mTRE4 (the mutated TRE4) co‐transfected with THRA or THRB showed significantly decreased luciferase activity (Figure 6K), suggesting that TRE4 might be the functional putative TRE. To further confirm this finding, TRE4 or mTRE4 and TR isoforms were co‐transfected. As anticipated, the WT TRE4 exhibited a dose‐dependent response to TRs (Figure S6F), whereas mTRE4 did not (Figure 6L), further confirming that TRs regulate COL11A2 via the COL11A2‐TRE4.

Collectively, TH may promote astrocyte differentiation and morphological development via TR‐dependent regulation of COL11A2.

### 2.7 COL11A2 Dysregulation Impairs Astrocyte Differentiation Across Mice, Pigs, and Humans

**FIGURE 7:**
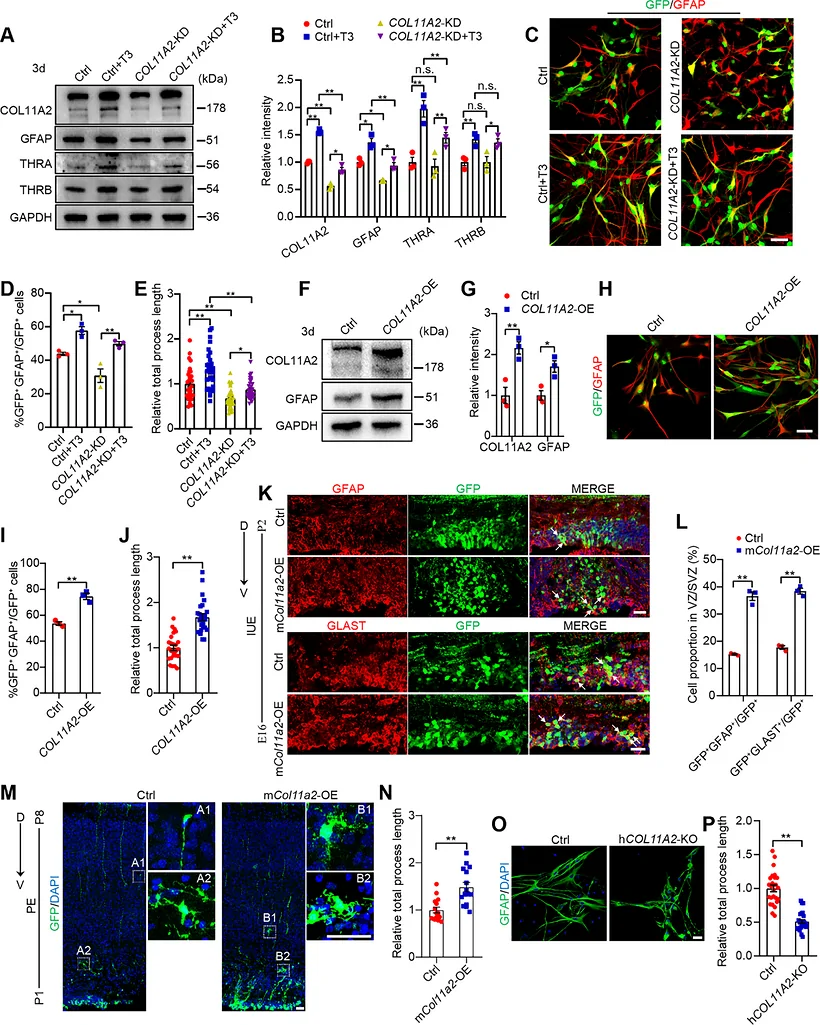
COL11A2 regulates astrocyte differentiation and morphological maturation. (A) Western blot analysis of the expression levels of COL11A2, THRA, THRB and GFAP in astrocytes derived from pNSCs under control or COL11A2 knockdown (KD) conditions, with or without T3 treatment. (B) Quantification of the protein levels in A (n = 3 biological replications). (C) Double immunofluorescence for GFAP and GFP in porcine astrocytes derived from pNSCs under control or COL11A2‐KD conditions, with or without T3 treatment. Scale bars, 100 µm. (D) Quantification of the proportion of GFAP^+^ astrocytes among GFP^+^ cells in C (n = 3 biological replications). (E) Quantification of total GFAP^+^ process length per GFP^+^GFAP^+^ cell in C (n = 3 biological replications, 30 cells per group). (F) Western blot analysis of the expression levels of GFAP in porcine astrocytes with COL11A2 overexpression (OE). (G) Quantification of the protein levels in F (n = 3 biological replications). (H) Double immunofluorescence for GFAP and GFP in porcine astrocytes derived from pNSCs with COL11A2 OE. Scale bars, 100 µm. (I) Quantification of the proportion of GFAP^+^ astrocytes among GFP^+^ cells in H (n = 3 biological replications). (J) Quantification of total GFAP^+^/GFP^+^ process length in H (n = 3 biological replications, 25 cells per group). (K) Double immunofluorescence for GFAP and GFP, or GLAST and GFP, in the brains of mice at P2 after m*Col11a2* OE by in utero electroporation (IUE) at E16. Scale bars, 50 µm. The arrows mark the GFAP^+^ GFP^+^ or GLAST^+^ GFP^+^ cells. (L) Quantification of the proportion of GFAP^+^ GFP^+^ or GLAST^+^ GFP^+^ cells among GFP^+^ cells in K (Ctrl, n = 3 brains; OE, n = 3 brains). (M) Immunofluorescence for GFP, combined with DAPI staining, in the brains of mice at P8 after m*Col11a2* OE by postnatal electroporation (PE) at P1. The areas within the boxes were magnified and are shown on the right. Scale bars, 50 µm. (N) Quantification of total process length in M (Ctrl, n = 3 brains; OE, n = 3 brains, 15 cells per group). (O) Immunofluorescence for GFAP, combined with DAPI staining, in human astrocytes with h*COL11A2* knockout (KO). Scale bars, 200 µm. (P) Quantification of total GFAP^+^ process length in O (n = 3 biological replications, 25 cells per group). All coronal sections were obtained from the right hemispheres of mice. D, dorsal; V, ventral. Data are shown as mean ± SEM. n.s., not significant; *P < 0.05; **P < 0.01 (two‐tailed t test or one‐way ANOVA followed by Holm–Sidak's multiple comparisons test).

To validate the essential role of COL11A2 in astrocyte differentiation, we utilized a shRNA lentivirus to knock down *COL11A2* in pNSCs (Figure S7A), and then differentiated the cells for 3 days. Western blot analysis showed that *COL11A2* knockdown (KD) led to a reduction in GFAP expression, while THRA and THRB levels remained unchanged (Figure 7A,B). After treating *COL11A2*‐KD astrocytes with 100 nM T3 for 24 h, GFAP expression increased, accompanied by elevated COL11A2, THRA, and THRB levels (Figure 7A,B). Consistent with the protein expression changes, immunostaining analysis showed marked reductions in both the proportion of GFAP^+^ cells among GFP^+^ cells and the total GFP^+^ process length of GFP^+^GFAP^+^ cells (Figure 7C–E). These morphological alterations were partially rescued by T3 treatment in *COL11A2*‐KD astrocytes (Figure 7C–E). Conversely, overexpression (OE) of *COL11A2* in pNSCs promoted GFAP expression (Figure 7F,G), increased the proportion of GFAP^+^ cells among GFP^+^ cells, and enhanced GFAP^+^ process extension (Figure 7H–J). These findings suggest a vital role of COL11A2 in porcine astrocyte differentiation and morphological maturation.

To evaluate the role of COL11A2 in astrocyte development in vivo, we conducted in utero electroporation (IUE) at various developmental stages in mice. We introduced m*Col11a2* OE plasmids through electroporation into the lateral ventricle of embryonic day 15 (E15) brains, and collected the brains 3 days later at E18. Immunostaining revealed a significant increase in the number of cells co‐labeled with GFAP and GFP in m*Col11a2* OE brains (Figure S7B,C). We further performed m*Col11a2* OE by IUE at E16 and collected brains at P2. Immunostaining confirmed that m*Col11a2* OE increased the number of GFP^+^ cells expressing GLAST, GFAP, or ZBTB20 (Figure 7K,L; Figure S7D,E), indicating enhanced astrocyte differentiation in vivo. To assess astrocytic morphology during postnatal development, we overexpressed m*Col11a2* at P1 and measured developmental progress within 7 days at P8, a time point when cortical astrocytes exhibit more complex morphological features [50]. We observed that the morphology of astrocytes in the cerebral cortex of m*Col11a2* OE mice was more complex than that of the control group (Figure 7M,N; Figure S7F). Together, these results indicate that m*Col11a2* OE promotes astrocytic differentiation and morphological maturation in vivo.

To examine whether COL11A2 affects human astrocyte development, we knocked out *COL11A2* in WIBR3 human embryonic stem cells (hESCs) using CRISPR/Cas9 technology (Figure S7G,H) and assessed their capacity for astrocyte differentiation. *COL11A2* KO hESCs and control hESCs were differentiated into human neural precursor cells (hNPCs) for 7 days, and were then cultured in astrocyte differentiation medium for an additional 23 days to promote astrocyte formation. Astrocytes derived from *COL11A2* KO hESCs exhibited prominently shorter process lengths than those from control hESCs (Figure 7O,P). Taken together, these observations demonstrate an important and conserved role for COL11A2 in astrocyte differentiation and morphological maturation across pigs, mice, and humans.

## 3 Discussion

In the CH pig model, we observed cerebral atrophy, cortical hypogyrification, and subcortical white matter dysplasia with glial defects, recapitulating key neuropathological abnormalities reported in infants with CH [8, 9, 10, 11]. Interestingly, the results from pathological and scRNA‐seq analyses revealed more pronounced alterations in glial cells than in neurons under CH conditions. However, abnormalities in cerebral white matter, as well as glial defects such as hypomyelination, are rarely observed in neonatal CH rodents, which exhibit partially similar phenotypes of hypomyelination until young adult [51, 52]. This discrepancy could be attributed to TH deficiency occurring within specific time windows and differing brain developmental patterns across distinct mammals.

During prenatal development in placental mammals, TH acquisition initially relies on maternal transfer, followed by synthesis by the fetal thyroid gland [53]. The initiation of fetal thyroid function generally occurs around mid‐gestation in pigs and humans, while in rodents it happens during late gestation at E17.5‐E18, approximately corresponding to the completion of fetal cerebral cortical neurogenesis and the commencement of gliogenesis [54, 55, 56]. Consequently, fetal cerebral cortical neurogenesis is primarily regulated by maternal TH, while gliogenesis is regulated under the combined influence of both fetal and maternal TH [14, 57]. The developmental timeline for fetal brain linked to different sources of TH may explain why early maternal TH deficiency in rodent models leads to notable deficiencies in fetal cerebral neuronal progenitor proliferation, migration, and aberrant neuronal development [58, 59, 60], whereas CH models (fetal TH deficiency) do not yield similar effects [20]. These studies help explain why glial cells were primarily affected in the CH pig model.

Additionally, brain growth spurts, corresponding to periods of glial cell multiplication [61], are distinct across rodents, pigs, and humans. In pigs and humans, the brain growth spurt initiates prenatally around mid‐gestation and continues into early postnatal stages, whereas in mice and rats, it starts postnatally [29, 30, 62]. Furthermore, brain growth spurts in pigs and humans coincide with in‐utero cortical primary gyrification [63, 64, 65, 66], potentially explaining the cortical hypogyrification observed in CH piglets, similar to findings in human infants. Our study is the first to demonstrate in a gyrencephalic large‐animal model that severe TH deficiency is associated with impaired cerebral gyrification, recapitulating clinical observations in CH patients. Together with our pathological and single‐cell transcriptomic findings, these developmental differences provide experimental support for the preferential vulnerability of glial development to TH deficiency under CH conditions. In line with previous studies suggesting that gliogenesis contributes to cerebral gyrification [67, 68], our findings indicate that disrupted glial development may represent an important component of CH‐associated cortical malformation.

Astrocytes, among the most abundant types of glial cells, serve a dual role as both mediators of TH metabolism and targets of TH signaling in the brain [13]. However, much remains unknown about the underlying mechanisms linking TH signaling and astrocyte development. scRNA‐seq technology offers the opportunity to systematically investigate dysregulated molecular pathways in cell‐type‐specific alterations at single‐cell resolution. Our scRNA‐seq analysis revealed that TH effectors were highly expressed and dysregulated in astrocytes. Notably, *COL11A2* was preferentially enriched in a differentiated astrocyte state (Astro‐2) along the astrocyte differentiation trajectory, and this state was markedly depleted in CH brains, suggesting that COL11A2 represents a state‐associated molecular signature of this astrocyte subpopulation. These findings prompted us to further investigate the regulatory relationship between TH signaling and COL11A2.

COL11A2 belongs to the collagen family, which comprises 29 members and serves as a vital component of the extracellular matrix (ECM) [48, 69]. Collagens serve as structural elements, influencing the mechanical properties, organization, and shape of various tissues, while also regulating cell behaviors such as proliferation, migration, and differentiation [48, 69]. Although previous studies have associated COL1A2 with cortical folding and COL3A1 with cortical lamination [70, 71], these findings have primarily focused on neuronal development. In contrast, the roles of collagen‐related genes in glial cells, especially astrocytes, remain largely unexplored.

The ECM is recognized for its potential impact on glial development, particularly astrocyte specification and morphological maturation [72, 73, 74], yet the specific involvement of collagen genes in this process remains poorly understood. RNA‐seq analysis of various mouse brain and non‐brain tissues revealed that *COL11A2* is expressed in astrocytes [75]. Moreover, transcriptomic studies have reported altered *COL11A2* expression in the P14 rat cerebral cortex under hypothyroid conditions [76, 77]. In addition, *COL11A2* was detected in a previously reported ChAP‐seq dataset analyzing genome‐wide T3 target genes in TR‐overexpressing neural progenitor cells [47]. However, these findings reflect only a correlative relationship between TH signaling and *COL11A2* expression in neural cells and do not provide direct evidence of transcriptional regulation or functional impact on astrocyte development.

In our study, we confirmed that COL11A2 is a downstream target of the TH/TRs axis. Functionally, we demonstrated that dysregulation of COL11A2 impairs astrocyte differentiation and morphological maturation. Importantly, this effect extends to human astrocytes, highlighting the translational relevance of the CH pig model. To our knowledge, this study provides the first experimental evidence that the TH/TRs axis regulates astrocyte differentiation via COL11A2 (Figure S8). Nevertheless, TH‐regulated glial development is likely multifactorial, and the downstream mechanisms by which COL11A2 regulates astrocyte differentiation and morphological maturation remain to be further investigated.

In summary, this study presents a suitable large animal model and establishes an association between endocrine regulation of glial development and cerebral gyrification. Our data demonstrate that the TH/TRs–COL11A2 axis regulates astrocyte differentiation and morphological maturation. However, the direct causal contribution of astrocyte differentiation‐state alterations to cortical hypogyrification remains to be determined, and long‐term in vivo rescue experiments are currently not feasible in this neonatal CH pig model. Future studies using temporally controlled or cell‐type‐targeted approaches will be required to directly test whether astrocyte differentiation defects causally contribute to CH‐associated cortical malformations. Additionally, the weak expression of COL11A2 in the oligodendrocyte lineage also raises the possibility of roles beyond astrocytes. These findings provide a foundation for exploring how CH disrupts glial development and offer insights into a broad spectrum of neurodevelopmental disorders associated with cortical malformations.

## 4 Experimental Section

### 4.1 Animals

The Hypo pigs and their littermate WT controls used in this study were all produced by crossing heterozygous male and female Bama miniature pigs carrying the *DUOX2 ^+/D409G^* mutation, which was generated in a large‐scale ENU mutagenesis screen at the Beijing Farm Animal Research Center [26]. Since neonatal Hypo pigs exhibited markedly reduced vitality and could not survive beyond birth, the brains used in this study were harvested at P0. P0 pig brains represent a perinatal developmental stage broadly aligned with human late prenatal to early postnatal brain development [78]. The ICR pregnant mice for IUE were purchased from Vital River Laboratories (Beijing, China). All experimental procedures of animals were approved by the Institutional Animal Care and Use Committee of the Institute of Zoology, Chinese Academy of Sciences, China (approval no. IOZ20180077).

### 4.2 Cell Lines and Culture

The HEK293T cells were cultured in Dulbecco's modified Eagle's medium (DMEM; Gibco) supplemented with 10% fetal bovine serum (FBS, Hyclone) and 1% penicillin/streptomycin (Gibco) in a humidified 5% CO2 incubator at 37°C.

The WIBR3 hESCs were maintained on Matrigel (Corning)‐coated 6‐well cell culture plates (Corning) with Essential 8 medium (Gibco) at 37°C in an incubator with 5% CO_2_. The medium was changed every day. Cells were passaged every four days using a 3‐min incubation with 0.5 mM EDTA followed by dissociation into small clumps.

### 4.3 Primary Cells and Culture

pNSCs were isolated as described previously with slight modifications [79]. In brief, we excised whole brains from newborn piglets immediately after sacrifice, placed them in 20 mL fresh PBS, and then dissected them. First, the cerebellum and olfactory bulb were removed with dissecting forceps to obtain the cerebral cortex. Next, a midline incision was performed between the hemispheres. The meninges were then pulled and removed from the cortex hemisphere. Part of the cerebral cortex tissue was extracted, minced, and then transferred into a sterile 15 mL centrifuge tube. The minced tissues were enzymatically digested with 3 mL 0.25% trypsin and incubated at 37°C for 30 min with gentle shaking every 10 min. The cell suspension was centrifuged and resuspended in porcine NSC medium by vigorous pipetting. The cells were then plated in 6‐ or 24‐well plates coated with poly‐D‐lysine (10 µg/mL, Sigma) and laminin (10 µg/mL, Sigma) for 4 h at 37°C. The porcine NSC medium was composed of DMEM/F12 medium (Gibco) supplemented with 40 ng/mL EGF (Gibco), 40 ng/mL bFGF (Gibco), 1% N2 supplement (Gibco), 2% B27 supplement (Gibco), 1% glutamine (Gibco), 1% penicillin/streptomycin, and 1.5 ng/mL human LIF (hLIF, Millipore). The medium was changed 2 days after plating and every day thereafter.

### 4.4 Cresyl Violet Staining

The right hemisphere of each brain was fixed in 4% neutral buffered paraformaldehyde (PFA) for histological analysis. After dehydrating in a series of graded alcohols (70%–100%), the fixed tissues were embedded in paraffin and sliced to a thickness of 5 µm using a rotary microtome (Leica). The sections were deparaffinized and rehydrated using xylene and graded alcohols, and then stained with 0.1% cresyl violet solution (Sigma) according to the manufacturer's protocol. The sections were then visualized using a NanoZoomer S210 Digital slide scanner (Hamamatsu Photonics).

### 4.5 Gyrification Index Measurement

The gyrification index (GI) was measured as previously described [35]. Briefly, GI was calculated as the ratio of the complete cortical contour length, which follows the sulcal and gyral folds, to the outer cortical contour length. Measurements were performed on cresyl violet‐stained coronal brain sections using ImageJ software.

To assess regional variability along the rostrocaudal axis, three anatomically matched coronal sections were selected from the right hemisphere of each brain, corresponding to rostral, middle, and caudal regions. For each section, the complete cortical contour and the outer cortical contour were manually traced, and GI was calculated separately.

### 4.6 T3 Content Measurement

Weighed aliquots of brains from WT and Hypo individuals were finely minced and homogenized individually in saline using a tissue grinder. The homogenate was centrifuged at 3500 rpm for 20 min in a refrigerated centrifuge set at 4°C, and the supernatant was collected. T3 content was subsequently measured by radioimmunoassay following extraction of T3 in Beijing Kangjia Hongyuan Biotechnology Co., Ltd.

### 4.7 Immunofluorescence Staining

For paraffin‐embedded porcine brain sections, heat‐induced antigen retrieval was performed with citrate buffer (pH 6.0) for 15 min after deparaffinization and rehydration, followed by permeabilization with 0.3% Triton X‐100 in phosphate‐buffered saline (PBS) for 1 h. Mouse brains were fixed in 4% PFA and dehydrated in 30% sucrose. Fifteen‐micrometer brain slices were obtained using the freezing microtome (Leica). The frozen sections or cells used in this study were fixed in 4% PFA for 20 min and washed three times in PBS containing 0.1% Triton X‐100.

The sections or cells were incubated with 5% bovine serum albumin (BSA) blocking buffer for 1 h and further incubated with primary antibodies overnight at 4°C and fluorescence‐labeled secondary antibodies at room temperature for 1 h. Nuclei were counterstained with 4′,6‐diamidino‐2‐phenylindole (DAPI, Thermo Fisher Scientific). The slides were then visualized using a Zeiss LSM880 confocal laser scanning microscope or PerkinElmer Vectra Polaris automated quantitative pathology imaging system. The antibodies used for immunostaining are listed in Table S4.

### 4.8 Preparation of Cell Suspensions of Tissue Samples

The single‐cell suspension was obtained using the Adult Brain Dissociation Kit (Miltenyi), according to the manufacturer's protocol with minor modifications. In brief, the cerebral cortex and the adjacent subcortical white matter from all four lobes were dissected from the brain of one Hypo pig and its WT littermate. The dissected tissue was transferred to an enzyme mixture and subjected to mechanical dissociation using the gentleMACS Dissociators, followed by incubation at 37°C for 30 min. The cell suspension was filtered to eliminate tissue debris and then centrifuged at 3,000 g for 10 min. The cells were resuspended and subjected to another centrifugation step (1000 g, 10 min) to remove any remaining cell debris. Finally, the cells underwent an incubation process for red blood cell removal.

### 4.9 scRNA‐seq Library Preparation and Sequencing

The cell suspension was loaded onto the Chromium Single Cell Controller (10x Genomics) to generate single‐cell gel beads in emulsion (GEMs) using the Chromium Single Cell 3′ GEM Library & Gel Bead Kit v3 (10x Genomics) and Chromium Single Cell B Chip Kit (10x Genomics), following the manufacturer's protocol. Approximately 10,000 captured cells per sample were lysed, and the released RNAs were barcoded through reverse transcription in individual GEMs. Reverse transcription was performed on an S1000TM Touch Thermal Cycler (Bio‐Rad). The cDNA was subsequently amplified, and its quality was assessed using an Agilent 4200. The cDNA libraries were sequenced on an Illumina NovaSeq 6000 sequencer, employing a paired‐end 150 bp (PE150) reading strategy (conducted by CapitalBio Technology, Beijing).

### 4.10 Data Processing and Analysis of scRNA‐Seq Data

The raw data were processed using Cell Ranger (version 6.0.0, 10x Genomics) with the Sscrofa 11.1 reference genome to generate the digital gene expression matrix. Subsequently, the output expression matrix from Cell Ranger underwent processing with Seurat (version 4.3.2, https://satijalab.org/seurat/) for filtering, data normalization, dimensionality reduction, clustering, and gene differential expression analysis.

Cells with fewer than 500 or more than 5,000 genes, and fewer than 1,000 unique molecular identifiers (UMI), or a mitochondrial gene ratio of more than 15% were excluded. It is noteworthy that the number of captured cells in the Hypo‐TL sample exceeded 20,000. To ensure comparability with other samples, we set the parameter ‘set.seed()’ with the random number ‘random_num <‐ sample(1:21, 798, 10000)’, randomly selecting 10,000 cells for subsequent analysis.

After quality control, downstream bioinformatic analyses were performed following the online pipeline (https://satijalab.org/seurat/). Cells were visualized using a 2‐dimensional *t*‐SNE algorithm. DEG analysis was conducted with the “FindMarkers” function of Seurat between WT and Hypo groups using the Wilcox test. Only those with adjusted *P* values < 0.05 and |logFC| > 0.5 were identified as DEGs. The clusterProfiler (version 4.6.2) package was used for GO term enrichment analysis and visualized using the ggplot2 package (version 3.4.2). GSVA package (version 1.50.5) was used to assign pathway activity estimates to individual cell types using standard settings. CytoTRACE2 (version 1.0.0) was used to predict the relative differentiation state of cell subpopulations [80]. Monocle2 (version 2.26.0) was employed to infer developmental trajectories and estimate pseudotime following the default settings [81].

### 4.11 qRT‐PCR

Total RNA was isolated from tissues or cells using Trizol (Thermo Fisher Scientific) and reverse transcribed to cDNA using a PrimeScript RT Reagent Kit with gDNA Eraser (Takara). qRT‐PCR was performed on a QuantStudio 6 Flex real‐time PCR system (Applied Biosystems) using a SYBR Premix EX Taq Kit (Takara). The relative mRNA expression levels of the target genes were calculated as the fold changes of the threshold cycle (Ct) value relative to the reference using the 2^−ΔΔCt^ method. The sequences of the primers used are listed in Table S5.

### 4.12 Western Blot

Tissues and cells were homogenized and lysed in T‐PER tissue protein extraction reagent (Thermo Fisher Scientific) and RIPA lysis buffer (Beyotime) supplemented with protease inhibitor cocktail (Thermo Fisher Scientific), respectively. The concentration of protein was measured by a BCA kit (Solarbio). Lysates were mixed with an equal volume of 2 × Laemmli sample buffer (Bio‐Rad), and boiled for 10 min. The protein extracts were electrophoresed on 8% SDS‐PAGE gels and were transferred to PVDF membranes (Bio‐Rad). Membranes were blocked with 5% skimmed milk for 2 h at room temperature and incubated overnight at 4°C with primary antibodies. The detection was carried out with a horseradish peroxidase–conjugated (HRP) secondary antibody and visualized with an enhanced chemiluminescence (ECL) kit (Thermo Fisher Scientific) using a Tanon 5200 multi‐chemiluminescent imaging system. The quantification was performed using ImageJ software. The antibodies used for the western blot are listed in Table S4.

### 4.13 RNA Fluorescence In Situ Hybridization

RNA fluorescence in situ hybridization for *COL11A2* was performed manually using a PinpoRNA RNA in situ hybridization detection kit (Pinpoease Biotech Co.Ltd.) according to the manufacturer's instructions. The *COL11A2* probe was designed to target the 155–1520 nucleotide region of porcine *COL11A2* mRNA. A *DapB* probe targeting the bacterial *DapB* gene was used as a negative control.

Briefly, 5‐µm‐thick paraffin sections were deparaffinized with xylene and graded alcohols. The sections were incubated in pretreatment solution A at room temperature for 10 min, washed with ultrapure water, and boiled in 1× pre‐reaction solution B containing citrate buffer at 95°C–100°C for 15 min. After immersion in alcohol for 3 min and air drying, hydrophobic barriers were drawn around the tissue sections. The sections were then digested with protease at 40°C for 10 min and hybridized with the *COL11A2* probe in probe hybridization buffer at 40°C for 2 h. After washing, signal amplification was performed by sequential incubation with reaction solution 1, reaction solution 2, and HRP‐containing reaction solution 3 at 40°C for 25, 15, and 15 min, respectively. Fluorescent signals were developed using a tyramide signal amplification reaction. The sections were then subjected to immunofluorescence staining with anti‐S100β antibody and counterstained with DAPI.

### 4.14 Generation of Porcine Astrocytes From pNSCs

For porcine astrocyte differentiation, the culture medium was changed to the glial differentiation medium after pNSCs reached 70%–80% confluency. The differentiation medium was composed of DMEM/F12 medium (Gibco) supplemented with 1% N2 supplement (Gibco), 2% B27 supplement (Gibco), and 1% FBS (Gibco). The culture medium was refreshed two days after the cells were plated.

### 4.15 Generation of hNPCs and Astrocytes From hESCs

hNPCs were generated from hESCs as previously described [82]. Briefly, hESCs reached 70%–80% confluency in 6‐well plates, the medium was replaced with the hNPCs differentiation medium consisting of DMEM/F12 medium (Gibco), 50% Neurobasal medium (Gibco), 1% GlutaMAX (Gibco), 2% B27 supplement minus vitamin A (Gibco), 1% N2 supplement (Gibco), 2 × 10^−6^ M dorsomorphin (Selleck), 3 × 10^−6^ M SB431542 (Tocris), 4 × 10^−6^ M CHIR99021 (Stemgent), 0.1 × 10^−6^ M compound E (EMD Chemicals), and 10 ng/ mL human LIF (Millipore). After 7 days, the cells were digested into single cells with Accutase (Invitrogen) and cultured on 6‐well plates pre‐coated with Matrigel. The medium was changed to hNPCs maintenance medium composed of DMEM/F12 medium, 50% Neurobasal medium (Gibco), 1% GlutaMAX (Gibco), 2% B27 supplement minus vitamin A (Gibco), 1% N2 supplement (Gibco), 2 × 10^−6^ M SB431542 (Tocris), 3 × 10^−6^ M CHIR99021 (Stemgent), and 10 ng/mL hLIF (Millipore).

For human astrocyte differentiation, the transition from NPC differentiation medium to astrocyte induction medium occurred when hNPCs reached around 80% confluence, typically 2–3 days after plating. The astrocyte differentiation medium was composed of DMEM/F12 medium containing 2% B27 supplement, 1% N2 supplement, 10 ng/mL BDNF (Peprotech), and 10 ng/mL GDNF (Peprotech). 1 µg/mL Laminin (Sigma) was added after 2 days of culture. 10 ng/mL hLIF or 10% FBS (Gibco) [83] was added after 4 days, and the medium was changed every two days.

### 4.16 ELISA Assay

Astrocytes were cultured in medium for 6 days, followed by 100 nM T3 (Sigma) treatment for 24 h. The supernatants from cultures were collected and measured with the Porcine COL11A2 ELISA Kit (JONLN).

### 4.17 Plasmid Construction

The ORFs of porcine *THRA* and *THRB* were cloned into the pcDNA3.1 vector to obtain pcDNA‐TR. Promoter regions containing the TREs of *COL11A2* were linked to the promoter region of the firefly luciferase gene carried in the pGL3‐Basic vector. The mutated TRE plasmids were constructed according to the MEGAWHOP protocol. Plasmid PCR amplification was performed using the KOD‐plus‐Neo DNA polymerase Kit (TOYOBO). The full‐length sequences of porcine *COL11A2* and mouse *Col11a2* (obtained from Changsha Youbio Biological Company) were cloned into the pCDH‐CMV‐MCS‐EF1‐copGFP vector with 3Flag‐tag. The complementary small hairpin RNA oligos for porcine *COL11A2* were synthesized and then annealed to double‐stranded DNA and cloned into the pSicoR‐GFP vector. The complementary single‐guide RNA oligos for human *COL11A2* were synthesized and then annealed to double‐stranded DNA and cloned into the PX458 vector. The sequences used for plasmid construction are listed in Table S5. Scrambled shRNA was used as the negative control for knockdown (KD) experiments, and empty vectors were used as the control for overexpression (OE) and knockout (KO) experiments.

### 4.18 Luciferase Assay

At 24–48 h after transfection, the luciferase assay was performed using a Dual‐Luciferase Reporter Assay System (Promega) according to the manufacturer's protocol. Firefly and renilla luciferase activities were measured on a GloMax 96 microplate luminometer (Promega).

### 4.19 Lentivirus Packaging and Infection

Lentiviral transfer plasmids, the envelope plasmids, and the packaging plasmids were co‐transfected into HEK293T cells by GenEscort^TM^I (Wisegen). The medium supernatant containing lentivirus was harvested at 24, 48, and 72 h post‐transfection. The medium supernatant was centrifuged at 3000 rpm for 5 min to eliminate cell debris. The lentivirus suspension was used to infect pNSCs with the addition of 2 µg/mL polybrene. Twelve hours later, the medium was changed to differentiation medium.

### 4.20 In Utero and Postnatal Electroporation

In utero electroporation was performed as described previously, with slight modifications [84, 85]. Briefly, pregnant mice were anesthetized with sodium pentobarbital by intraperitoneal injection, and the uterine horns were exposed. The recombinant plasmid together with Venus‐GFP plasmid at a molar ratio of 3:1 and 0.02% (w/v) fast green (Sigma) was mixed. Approximately 1–2 µL was gently microinjected into the embryonic lateral ventricle using a pulled glass micropipette. A total of 1.5 µg/µL pCDH‐CAG‐m*Col11a2*‐EF1‐copGFP and Venus‐GFP was used in the experiment. Each injected embryo within the uterus was placed between tweezer‐type electrodes. Five 50‐ms pulses with an interval of 950 ms at 45 V were given to E15 or E16 brains with a BTX ECM830 electroporator, and the brains were harvested 3 days later at E18 and 7 days later at P2, respectively. For P1 mice electroporation, we used five 50‐ms pulses with an interval of 950 ms at 100 V in the brains, and the brains were harvested 7 days later at P8.

### 4.21 h*COL11A2* Knockout in hESC

We knocked out h*COL11A2* using CRISPR/Cas9 gene editing. hESCs were digested using a 3‐min incubation with Accutase (STEMCELL Technologies). 1 × 10^6^ hESCs were electroporated with 10 µg sgRNA‐pSpCas9 (BB)‐2A‐GFP plasmid by the Lonza Nucleofector 2B device using Entranster‐E (eGreen). Transfected cells were seeded on a Matrigel‐coated 6‐well cell culture plate in Essential 8 medium containing 10 µM ROCK inhibitor Y27632 (Selleck). After 24 h, the medium was changed to standard Essential 8 medium. Three days later, GFP‐positive cells sorted by fluorescence‐activated cell sorter (FACS) were seeded onto a Matrigel‐coated 6‐well cell culture plate at a density of 500 cells per well. After 7 days, the cells were cloned for culture and PCR identification.

### 4.22 Statistical Analyses

Statistical analyses were performed using GraphPad Prism 8 software. An unpaired two‐tailed t‐test (for 2 groups) and one‐way ANOVA followed by Holm–Sidak's multiple comparisons test were applied for parametric data involving multiple groups. The data are presented as the mean ± SEM. A *P* value of < 0.05 was considered statistically significant. * and ** indicate *P* < 0.05 and *P* < 0.01, respectively.

## Author Contributions

J.Z., J.J., Y.W., and Y.Z. conceived the study. Y.Z. and H.M. designed the experiments. Y.Z., X.W., H.M., P.L., S.T., and J.C. performed the experiments and analyzed the data. R.Z., P.L., R.G., Y.Y., Z.Y., and G.Q. collected the samples. Y.Z. and H.M. wrote the manuscript, and J.Z., J.J., Y.W., P.L., and N.L. revised the manuscript. All authors read and approved the manuscript.

## Conflicts of Interest

The authors declare no conflicts of interest.

## Supporting information




**Supporting File**: advs77598‐sup‐0001‐SuppMat.pdf.

## Data Availability

The raw sequence data reported in this paper have been deposited in the Genome Sequence Archive under accession number CRA014704 [86] in National Genomics Data Center [87], China National Center for Bioinformation / Beijing Institute of Genomics, Chinese Academy of Sciences (GSA accession number: CRA014704 https://ngdc.cncb.ac.cn/gsa). Any additional information required to reanalyze the data reported in this paper is available from the corresponding author upon reasonable request.
