# Supplementary material for: TH/TRs–COL11A2 Axis Mediates Loss of a Differentiated Astrocyte State in Hypogyrified Brains
Source: Adv Sci (Weinh). 2026 Sep 16:e77598. Online ahead of print. doi: 10.1002/advs.77598 (PMC13583121; doi:10.1002/advs.77598)
Supplement: Supplementary file 1 [file ADVS-9999-e77598-s001.pdf]

Supporting Information

TH/TRs–*COL11A2* axis mediates loss of a differentiated astrocyte state in hypogyrified brains

Ying Zhang[1,2,7*], Xiao Wang[3,7], Hongyan Ma[1,2,4,7], Pengcheng Lyu[1,2], Shuangjie Tian[1,2,4], Ruojun Zong[1,2,4], Jiani Chen[5], Gang Ren[1,2,4], Yongjiang Yang[1,2,4], Zichen Yang[1,2,4], Guosong Qin[1,2], Naishi Li[6], Yanfang Wang[3*], Jianwei Jiao[1,2,4*] and Jianguo Zhao[1,2,4*]

[1]Key Laboratory of Organ Regeneration and Reconstruction, Institute of Zoology, Chinese Academy of Sciences, Beijing 100101, China.

[2]Beijing Institute for Stem Cell and Regenerative Medicine, Beijing 100101, China.

[3]State Key Laboratory of Animal Biotech Breeding, Institute of Animal Science, Chinese Academy of Agricultural Sciences (CAAS), Beijing 100193, China.

[4]University of Chinese Academy of Sciences, Beijing 100049, China.

[5]Department of Animal Genetics, Breeding and Reproduction, College of Animal Science, South China Agricultural University, Guangzhou, 510642, China.

[6]Department of Endocrinology, Key Laboratory of Endocrinology of National Health Commission, Peking Union Medical College Hospital, Chinese Academy of Medical Sciences and Peking Union Medical College, Beijing 100730, China.

[7]These authors contributed equally to this work.

*Correspondence: zhangy@ioz.ac.cn; wangyanfang@caas.cn; jwjiao@ioz.ac.cn; zhaojg@ioz.ac.cn

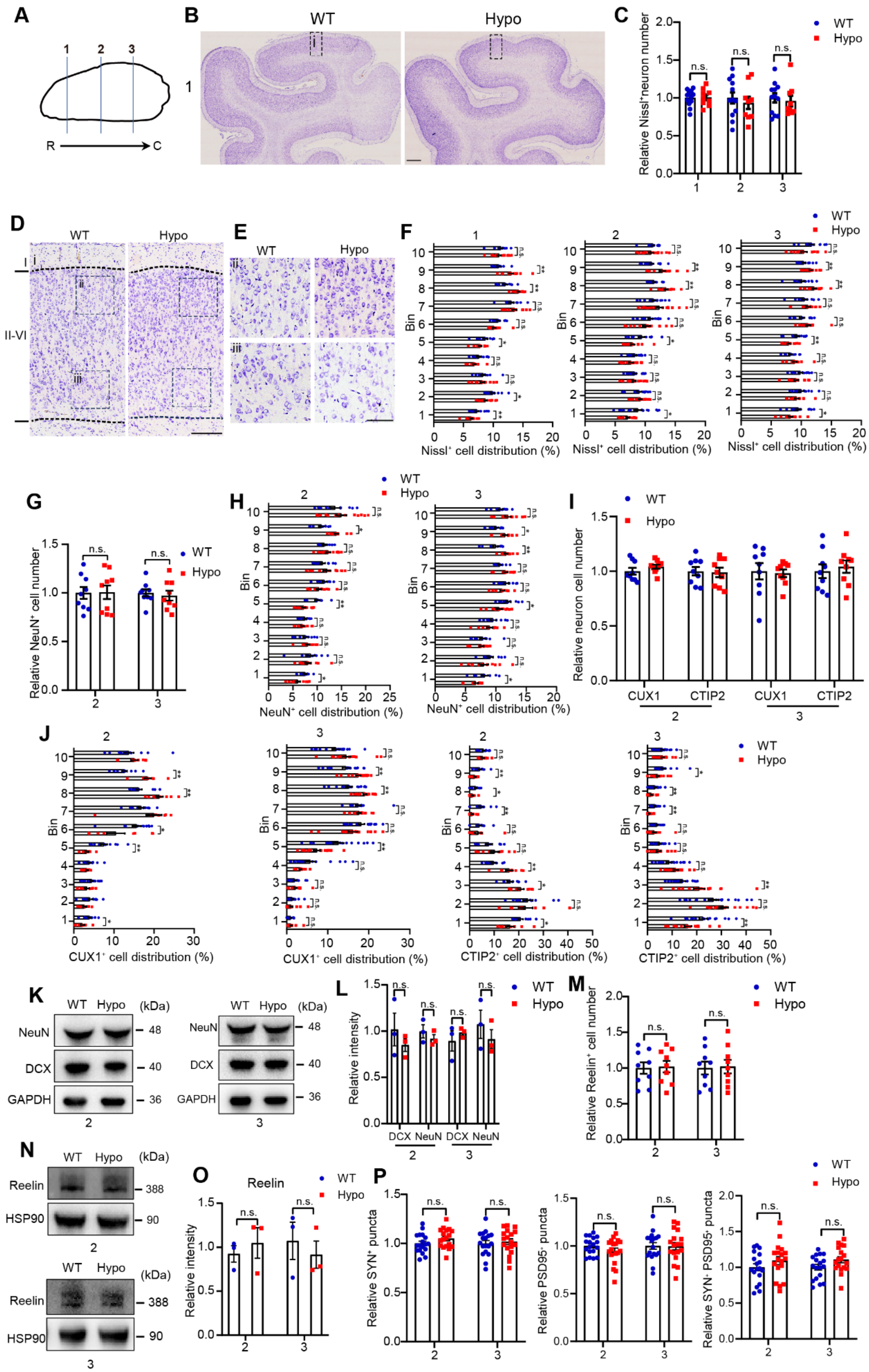
A
R
C
B
WT
Hypo
C
Relative Nissl+neuron number
n.s.
D
WT
Hypo
I
II-VI
E
F
Bin
Nissl+ cell distribution (%)
G
Relative NeuN+ cell number
H
NeuN+ cell distribution (%)
I
Relative neuron cell number
CUX1
CTIP2
J
CUX1+ cell distribution (%)
CTIP2+ cell distribution (%)
K
(kDa)
NeuN
DCX
GAPDH
L
Relative intensity
M
Relative Reelin+ cell number
N
Reelin
HSP90
O
Reelin
Relative intensity
P
Relative SYN+ puncta
Relative PSD95+ puncta
Relative SYN+ PSD95+ puncta

Figure S1. Analysis of cortical neurons in distinct regions of WT and Hypo pigs. (**A**) Diagram of coronal sections from the right hemispheres of pigs at three regions along the rostrocaudal axis. R, rostral; C, caudal. (**B**) Cresyl violet staining of the cortex of WT and Hypo pigs at the first region along the rostrocaudal axis. Scale bar, 1 mm. (**C**) Quantification of Nissl$^+$ neuron number throughout the cortex of **B** (WT, n = 4 pigs; Hypo, n = 3 pigs; 3 slices per pig). (**D**) Magnified views of the boxed areas in **B**. Scale bar, 250 μm. (**E**) Magnified views of the boxed areas in **D**. Scale bar, 100 μm. (**F**) Cortical bin analysis. The thickness of the cortical plate (CP), spanning from the outer border of layer II to the inner border of layer VI, was subdivided into 10 equal bins (bin 10, superficial, to bin 1, deep) for Nissl$^+$ neuron distribution quantification in three regions (WT, n = 4 pigs; Hypo, n = 3 pigs; 3-6 slices per pig). (**G**) Quantification of NeuN$^+$ neuron number in regions 2 and 3 (WT, n = 3 pigs; Hypo, n = 3 pigs; 3 slices per pig). (**H**) Cortical bin analysis in regions 2 and 3 for NeuN$^+$ neuron distribution quantification (WT, n = 3 pigs; Hypo, n = 3 pigs; 3-6 slices per pig). (**I**) Quantification of CUX1$^+$ and CTIP2$^+$ neuron number in regions 2 and 3 (WT, n = 3 pigs; Hypo, n = 3 pigs; 3 slices per pig). (**J**) Cortical bin analysis in regions 2 and 3 for CUX1$^+$ or CTIP2$^+$ neuron distribution quantification (WT, n = 3 pigs; Hypo, n = 3 pigs; 3-6 slices per pig). (**K**) Western blot analysis of NeuN and DCX protein levels in cortical tissues from regions 2 and 3. (**L**) Quantification of NeuN and DCX protein levels (WT, n = 3 pigs; Hypo, n = 3 pigs). (**M**) Quantification of Reelin$^+$ cell number in regions 2 and 3 (WT, n = 3 pigs; Hypo, n = 3 pigs; 3 slices per pig). (**N**) Western blot analysis of Reelin protein levels in cortical tissues from regions 2 and 3. (**O**) Quantification of Reelin protein levels (WT, n = 3 pigs; Hypo, n = 3 pigs). (**P**) Quantification of SYN$^+$ puncta, PSD95$^+$ puncta, and SYN$^+$PSD95$^+$ puncta in regions 2 and 3 (WT, n = 3 pigs; Hypo, n = 3 pigs; 6 slices per pig). Data are shown as mean ± SEM. n.s., not significant; *$P < 0.05$; **$P < 0.01$; two-tailed *t* test.

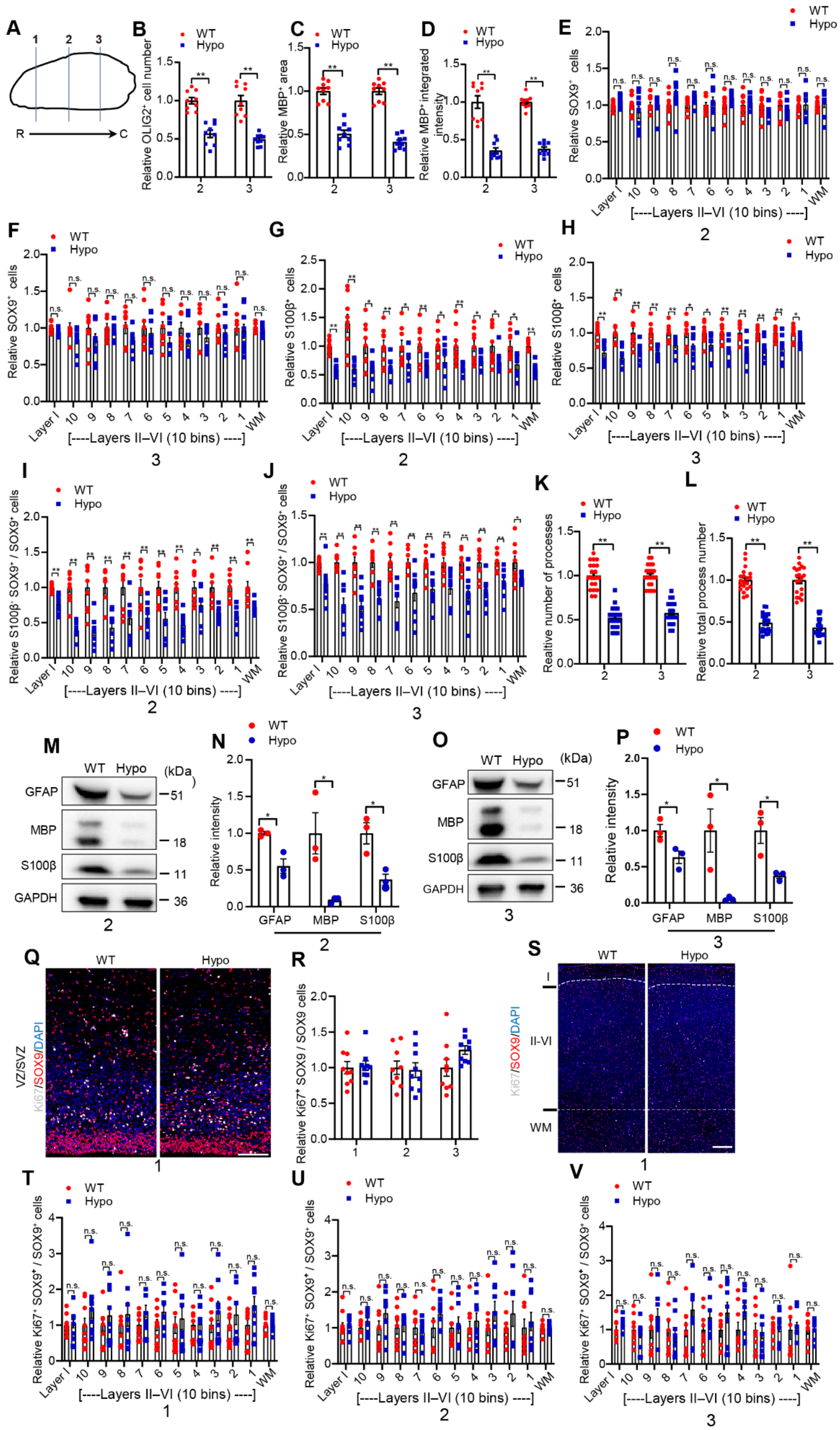

A
B
C
D
E
F
G
H
I
J
K
L
M
N
O
P
Q
R
S
T
U
V
WT
Hypo
R
C
Relative OLIG2⁺ cell number
Relative MBP⁺ area
Relative MBP⁺ integrated intensity
Relative SOX9⁺ cells
Relative S100β⁺ cells
Relative S100β⁺ SOX9⁺ / SOX9⁺ cells
Relative number of processes
Relative total process number
[----Layers II–VI (10 bins) ----]
Layer I
WM
GFAP
MBP
S100β
GAPDH
(kDa)
Relative intensity
VZ/SVZ
Ki67/SOX9/DAPI
Relative Ki67⁺ SOX9 / SOX9 cells
Relative Ki67⁺ SOX9⁺ / SOX9⁺ cells
I
II-VI
WM

Figure S2. Characterization of glial dysplasia in distinct regions of hypothyroid cortex and subcortical white matter. (**A**) Diagram of coronal sections from WT and Hypo right hemispheres at three regions along the rostrocaudal axis. (**B**) Quantification of $OLIG2^+$ cell number in regions 2 and 3 (WT, n = 3 pigs; Hypo, n = 3 pigs; 3 slices per pig). (**C-D**) Quantification of MBP^+ area or integrated intensity in regions 2 and 3 (WT, n = 3 pigs; Hypo, n = 3 pigs; 3 slices per pig). (**E-F**) Quantification of relative $SOX9^+$ cell number across layer **I**, 10 equal bins spanning cortical layers **II**-VI, and white matter across regions 2-3 (WT, n = 3 pigs; Hypo, n = 3 pigs; 3 slices per pig). (**G-H**) Quantification of relative $S100\beta^+$ cell number across layer **I**, 10 equal bins spanning cortical layers **II**-VI, and white matter across regions 2-3 (WT, n = 3 pigs; Hypo, n = 3 pigs; 3 slices per pig). (**I-J**) Quantification of $S100\beta^+SOX9^+$ cells among $SOX9^+$ cells across layer **I**, 10 equal bins spanning cortical layers **II**–VI, and white matter across regions 2-3 (WT, n = 3 pigs; Hypo, n = 3 pigs; 3 slices per pig). (**K-L**) Quantification of $GFAP^+$ process number and total $GFAP^+$ process length in subcortical white matter across regions 2-3 (WT, n = 3 pigs; Hypo, n = 3 pigs; 30 cells per group). (**M-P**) Western blot analysis and quantification of GFAP, MBP, and S100β protein levels in tissues across regions 2-3 (WT, n = 3 pigs; Hypo, n = 3 pigs). (**Q**) Representative images of $SOX9^+Ki67^+$ immunofluorescence in the VZ/SVZ of region 1. scale bar, 100 μm. (**R**) Quantification of $SOX9^+Ki67^+$ cells among $SOX9^+$ cells in the VZ/SVZ across regions 1-3 (WT, n = 3 pigs; Hypo, n = 3 pigs; 3 slices per pig). (**S**) Representative images of $SOX9^+Ki67^+$ immunofluorescence across cortex and white matter of region 1. scale bar, 200 μm. (**T-V**) Quantification of $SOX9^+Ki67^+$ cells across cortical layers (layer **I**, 10 equal bins spanning layers **II**-VI, and white matter) and white matter across regions 1-3 (WT, n = 3 pigs; Hypo, n = 3 pigs; 3 slices per pig). Data are shown as mean ± SEM; n.s., not significant; $^*P < 0.05$; $^{**}P < 0.01$; two-tailed *t* test.

A

Group	Brain region	No. cells	Mean UMI per cell	Mean gene per cell
WT	FL	9824	7590	2594
WT	PL	9295	6832	2382
WT	OL	10499	6977	2436
WT	TL	9424	6979	2399
Hypo	FL	8392	7423	2614
Hypo	PL	10319	7331	2556
Hypo	OL	8666	6959	2475
Hypo	TL	10000	6107	2337

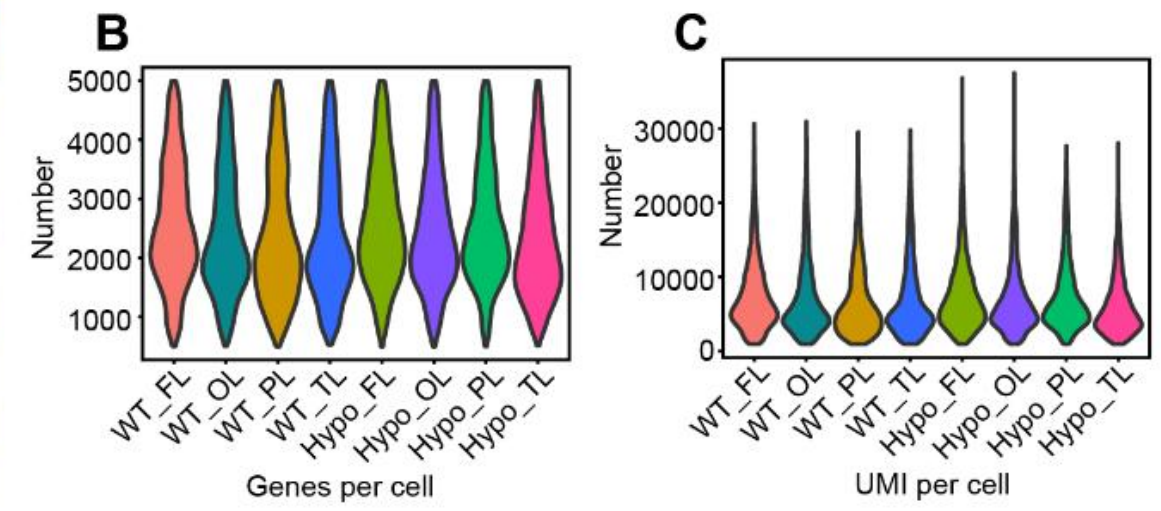

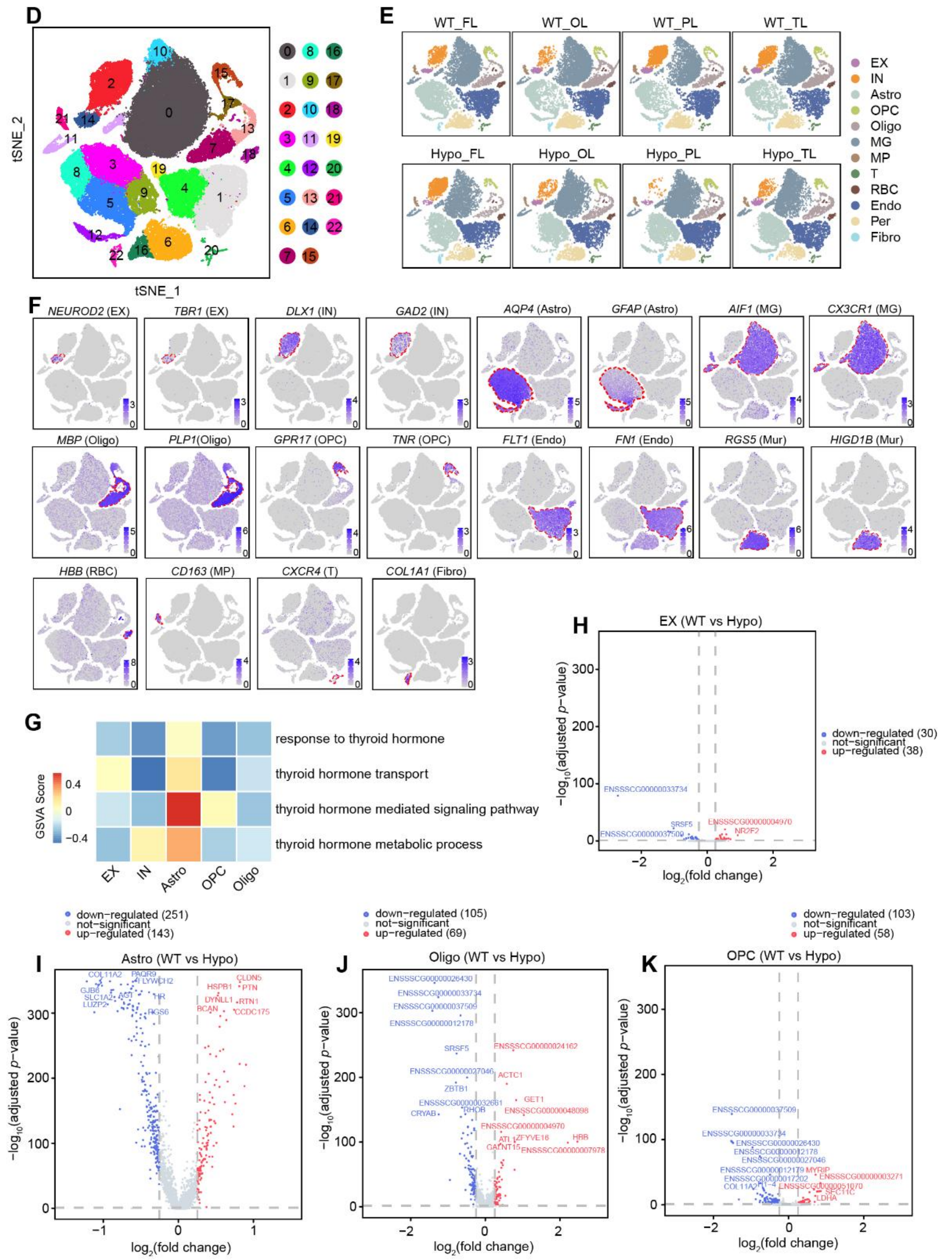

Figure S3. Quality assessment and cell type identification of scRNA-seq in pig brains. (**A**) Numbers of cells in each sample, mean UMI (unique molecular identifier) counts and mean genes in each cell after quality filtering. (**B**) Number of genes detected per cell of scRNA-seq quality metric. (**C**) Number of UMIs per cell of scRNA-seq quality metric. (**D**) *t*-SNE plot showing the initial cell clusters identified by scRNA-seq. (**E**) *t*-SNE visualization of cell-type distributions in distinct brain regions from WT and Hypo groups. (**F**) Expression levels of typical marker genes in distinct cells of brains using *t*-SNE plots. (**G**) Heatmap showing pathway activities scored per cell type by GSVA. (**H**) Volcano plot showing differentially expressed genes (DEGs) between WT and Hypo excitatory neurons (EX). (**I**) Volcano plot showing DEGs between WT and Hypo astrocytes (Astro). (**J**) Volcano plot showing DEGs between WT and Hypo oligodendrocytes (Oligo). (**K**) Volcano plot showing DEGs between WT and Hypo oligodendrocyte progenitor cells (OPC). DEGs were defined by $\log_2$(fold change) > 0.25 and adjusted *p*-value < 0.05.

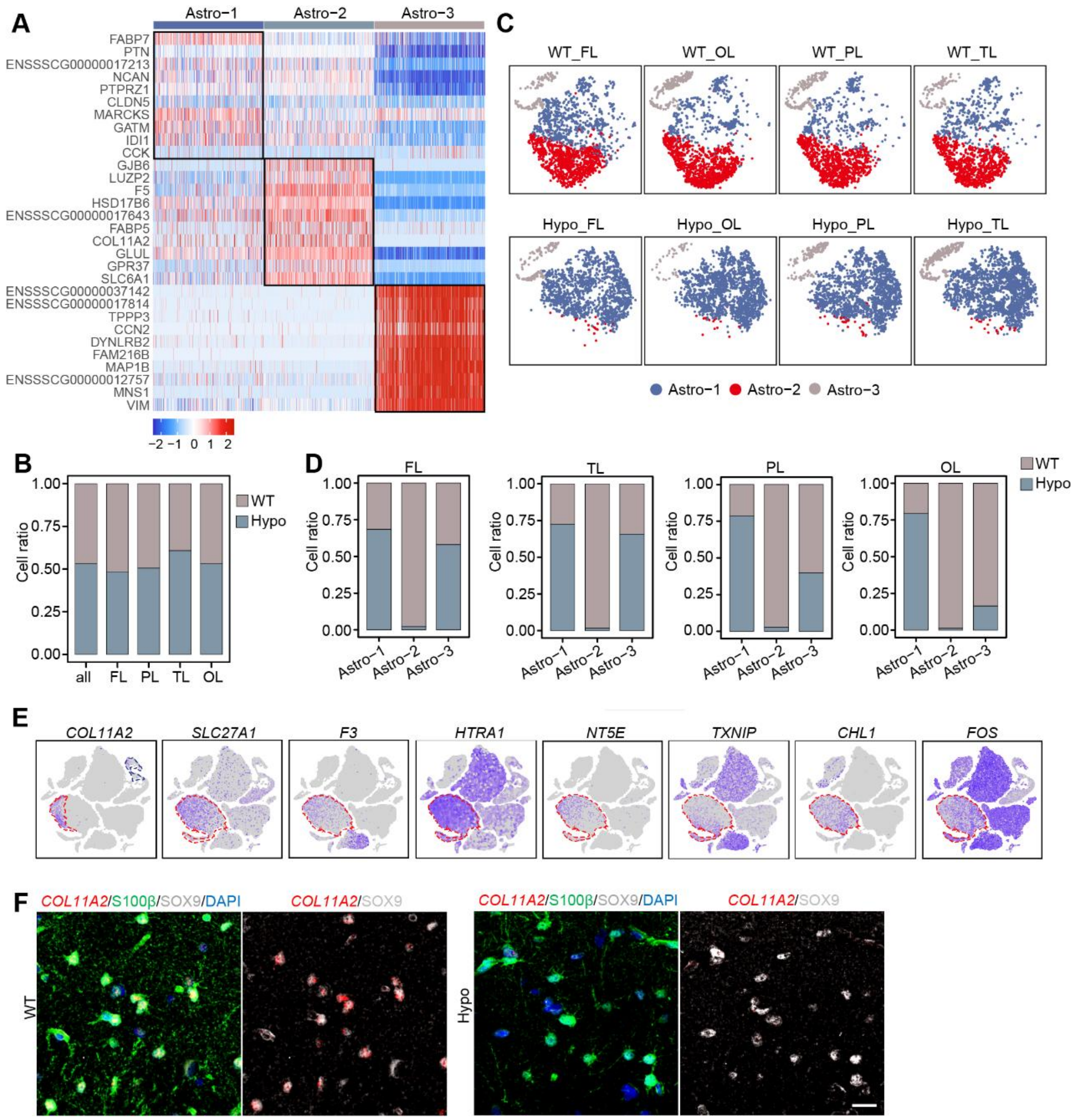

Figure S4. Analysis of the transcriptomic changes of astrocyte subpopulations in the Hypo brain. (**A**) Heatmap of differentially expressed genes in different astrocyte subtypes. (B) Cell ratios of WT and Hypo astrocytes in the combined dataset (all) and across different brain regions, including FL, PL, TL, and OL. (**C**) Distributions of astrocyte subpopulations in different regions of WT and Hypo brains using *t*-SNE plots. (**D**) Cell ratios of WT and Hypo astrocytes in various subtypes of different regions. (**E**) Expression distribution of *COL11A2*, *SLC27A1*, *F3*, *HTRA1*, *NT5E*, *TXNIP*, *CHL1* and *FOS* projected onto the *t*-SNE plot. The red dotted box delineates the astrocytes and the blue dotted box delineates the oligodendrocyte lineage. (**F**) RNA fluorescence

in situ hybridization for *COL11A2* combined with S100β and SOX9 immunofluorescence and DAPI staining in WT and Hypo pig brains. Scale bar, 20 μm.

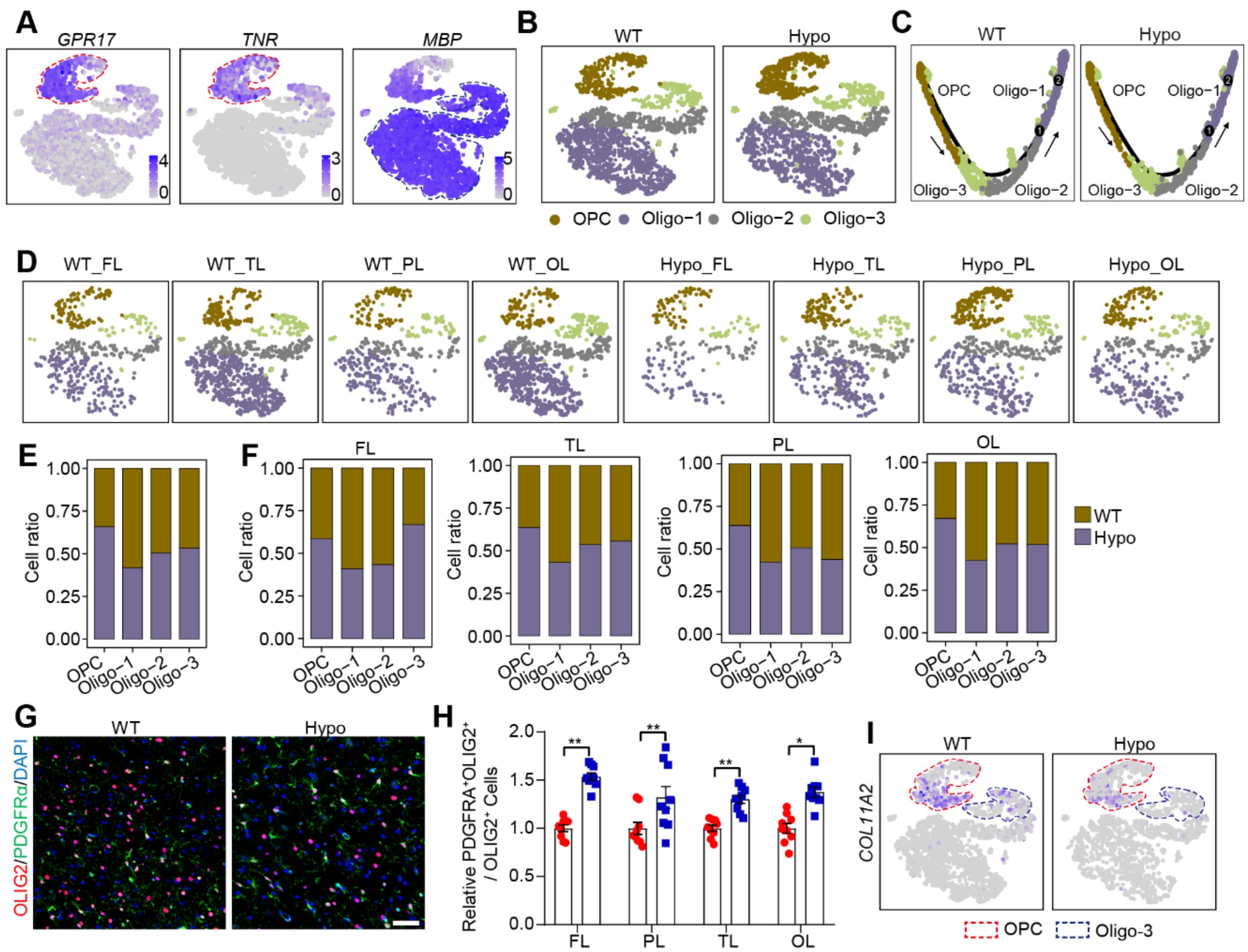

Figure S5. Analysis of the transcriptomic changes of the oligodendrocyte lineage in the Hypo brain. (**A**) Expression levels of typical marker genes in the oligodendrocyte lineage using *t*-SNE plots. (**B**) Distributions of oligodendrocyte lineage in WT and Hypo brains using *t*-SNE plots. Cells are colored by subtype. OPC, oligodendrocyte progenitor cells; Oligo-1, oligodendrocytes-1; Oligo-2, oligodendrocytes-2; Oligo-3, oligodendrocytes-3. (**C**) Pseudotime trajectory of the oligodendrocyte lineage. (**D**) Distributions of the oligodendrocyte lineage in different regions of WT and Hypo brains using *t*-SNE plot. (**E**) Cell ratios of the oligodendrocyte lineage in WT and Hypo brains across different subtypes. (**F**) Cell ratios of the oligodendrocyte lineage in WT and Hypo brains across subtypes and brain regions. (**G**) Representative immunofluorescence images of PDGFRα and OLIG2 in the subcortical white matter of WT and Hypo pigs. Scale bar, 50 μm. (**H**) Quantification of PDGFRα$^+$OLIG2$^+$ / OLIG2$^+$ cells across different regions (WT, n = 3 pigs; Hypo, n = 3 pigs; 3 slices per pig). (**I**) Expression levels of *COL11A2* in subpopulations of WT and Hypo

oligodendrocyte lineage using *t*-SNE plots. Data are shown as mean ± SEM, $^{*}P < 0.05$, $^{**}P < 0.01$ (two-tailed t test).

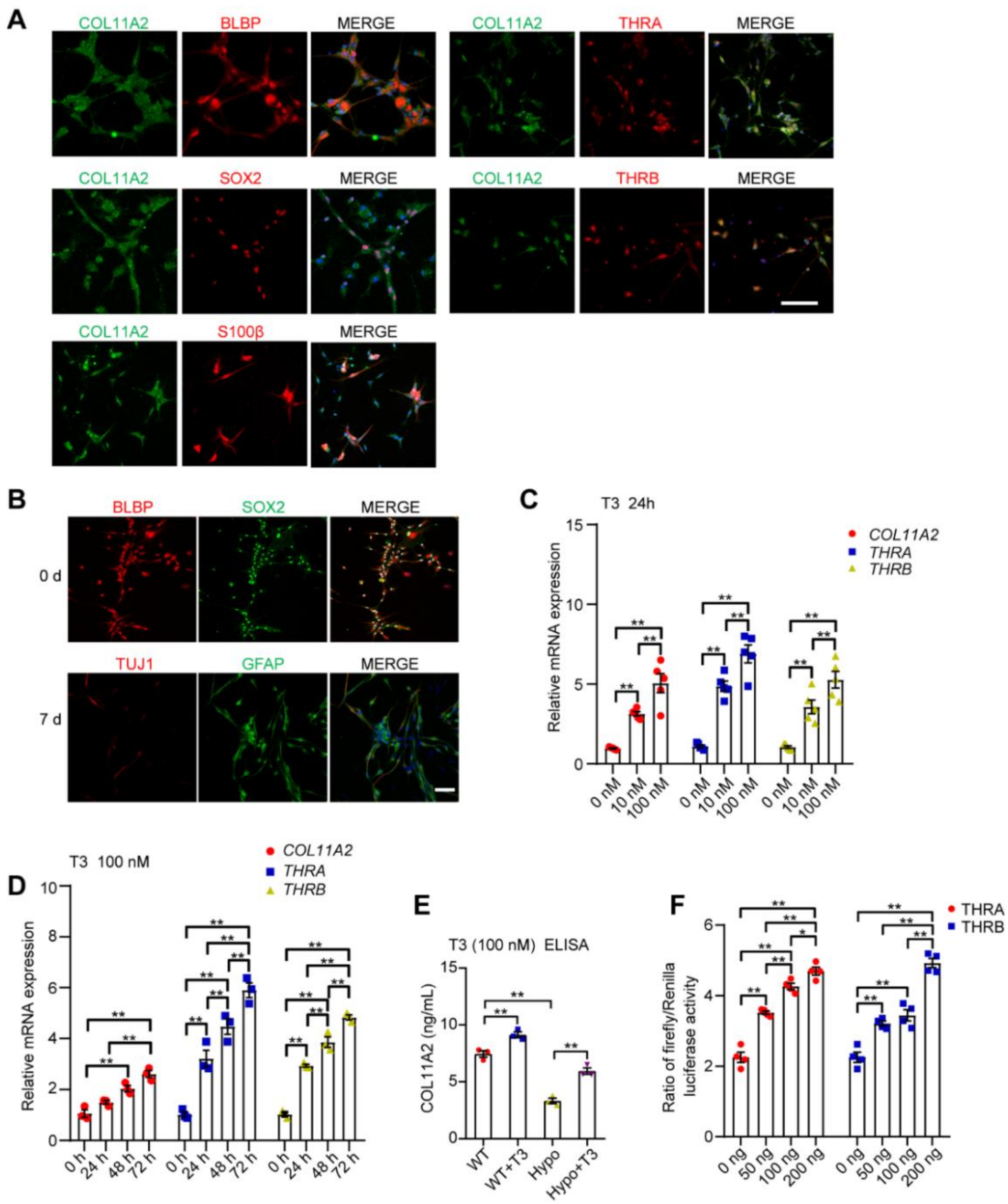

Figure S6. COL11A2 responds to the TH/TR axis regulation in porcine astrocytes. (**A**) Immunofluorescence co-localization analysis for COL11A2 with BLBP, SOX2, S100β, THRA and THRB in neural stem cells and astrocytes isolated from P0 brains of pigs. Scale bars, 50 μm. (**B**) Double immunofluorescence for TUJ1 and GFAP in BLBP$^+$ SOX2$^+$ neural stem cells cultured in differentiation medium for 7 days. Scale bars, 100 μm. (**C**) qRT-PCR analysis of *COL11A2*, *THRA* and *THRB* in pNSC-derived astrocytes treated with 0-100 nM T3 for 24 hours (n = 3 biological replications). (**D**) qRT-PCR analysis of *COL11A2*, *THRA* and *THRB* in pNSC-derived astrocytes treated with 100 nM T3 for 24-72 hours (n = 3 biological replications). (**E**) ELISA detection of COL11A2 secretion in culture medium from WT and Hypo pNSC-derived astrocytes, with or without T3 treatment (n = 3 biological replications). (**F**) Luciferase assay of TRE4 in response to distinct doses of TR isoforms (n = 3 biological replications).

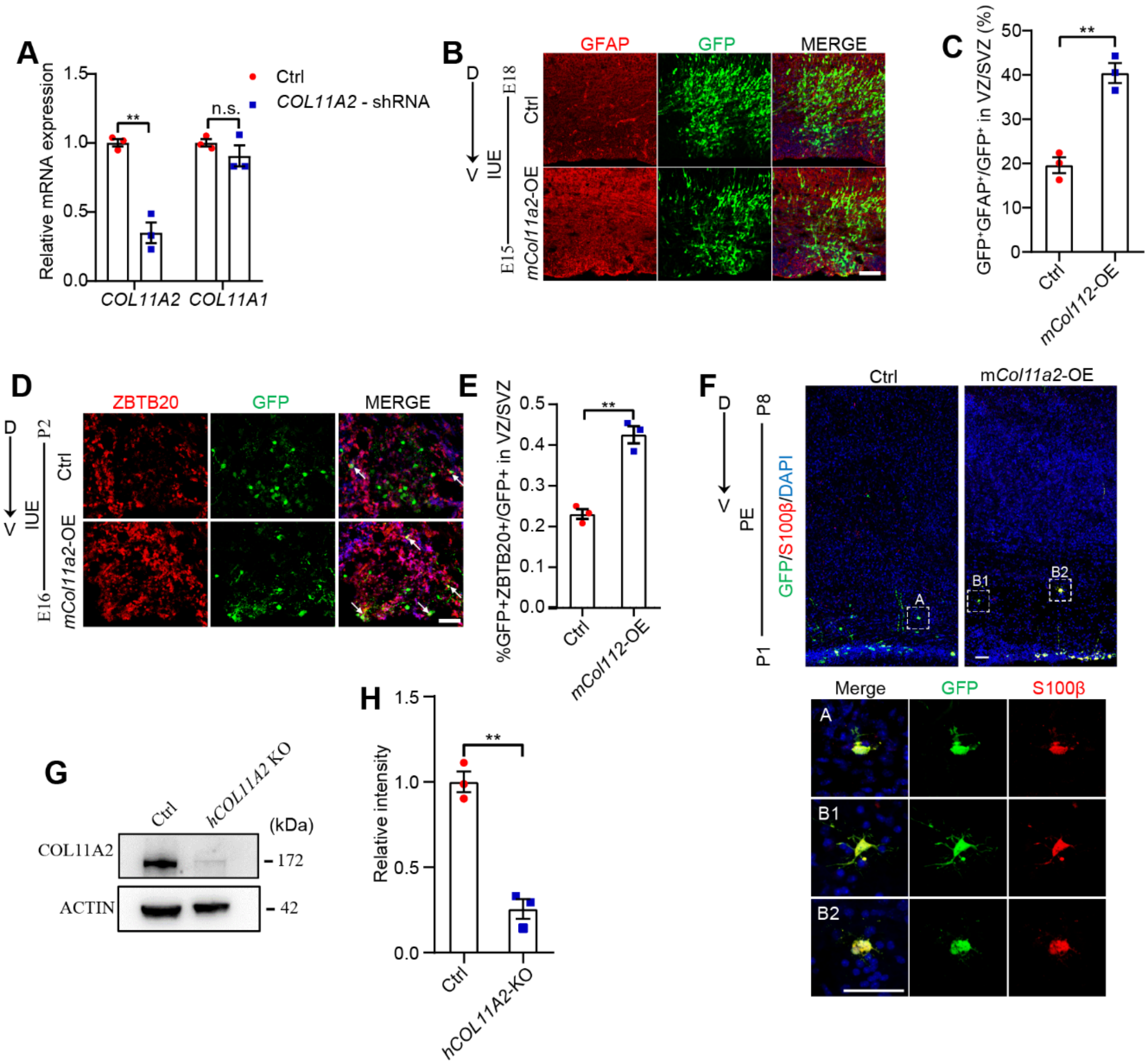

Figure S7. COL11A2 regulates astrocyte differentiation and morphological maturation. (**A**) Specificity analysis of *COL11A2*-shRNA knockdown. (**B**) Double immunofluorescence for GFAP and GFP in the brains of mice at E18 after in utero electroporation (IUE) at E15. Scale bars, 50 μm. (**C**) Quantification of the proportion of GFAP⁺ GFP⁺ cells among GFP⁺ cells in (**B**) (Ctrl, n = 3 brains; OE, n = 3 brains). (**D**) Double immunofluorescence for ZBTB20 and GFP in the brains of mice at P2 after in utero electroporation (IUE) at E16. Scale bars, 50 μm. (**E**) Quantification of the proportion of ZBTB20⁺ GFP⁺ cells among GFP⁺ cells in (**D**) (Ctrl, n = 3 brains; OE, n = 3 brains). (**F**) Double immunofluorescence for GFP and S100β, combined with DAPI staining, in the brains of mice at P8 after m*Col11a2* OE by postnatal electroporation (PE) at P1. The areas within the boxes were magnified and are shown on right. Scale bars, 50 μm. (**G**) Western blot analysis of the expression

levels of COL11A2 in hESCs with *COL11A2* knockout (KO). (**H**) Quantification of the protein levels in (**G**) (n = 3 biological replications). All coronal sections were obtained from the right hemispheres of mice. D, dorsal; V, ventral. Data are shown as mean ± SEM. n.s., not significant; $^{**}P$ < 0.01 (two-tailed t test).

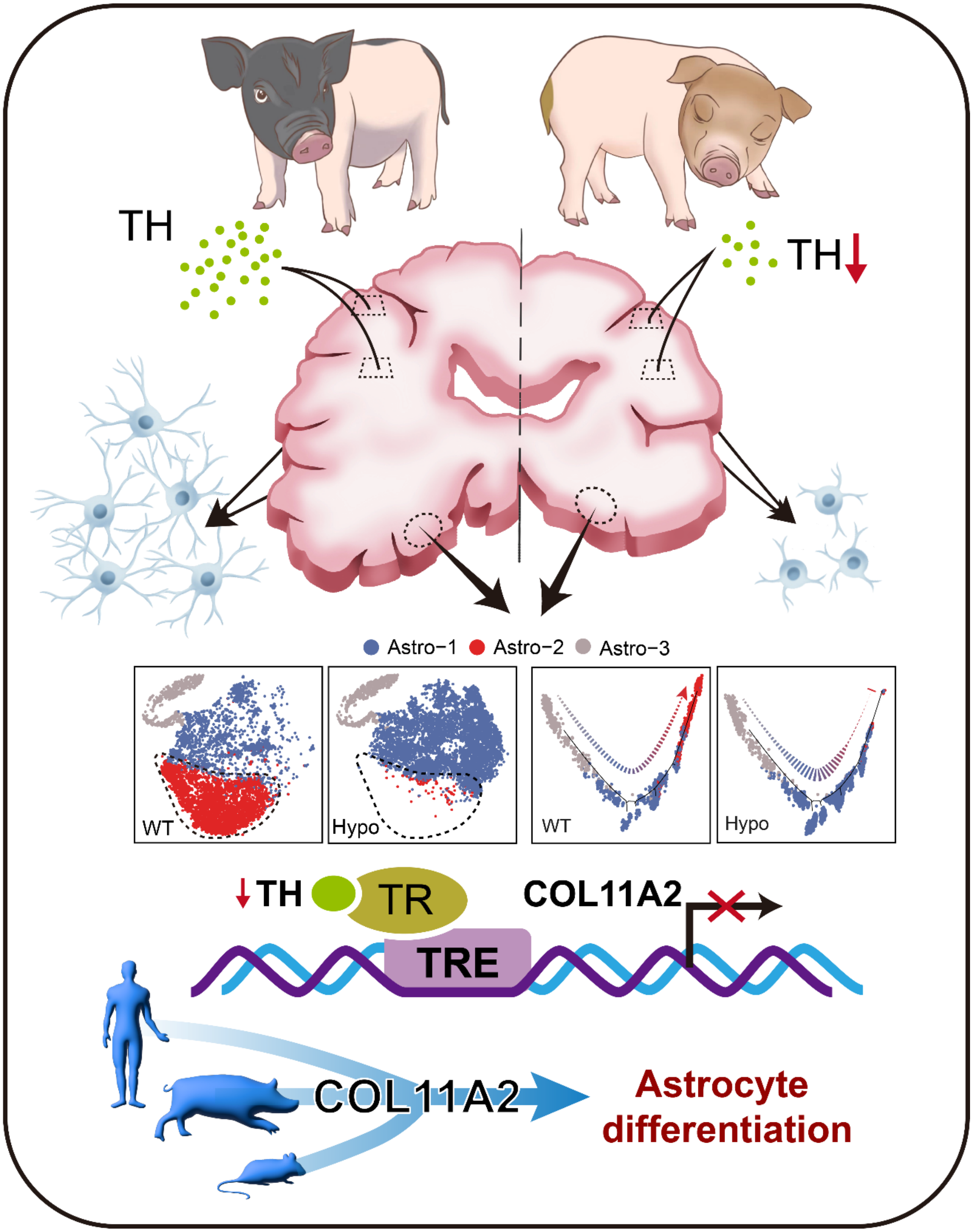

Figure S8. Model of TH regulating astrocyte differentiation via the TR-COL11A2 axis.

Table S1. Animal information for WT and Hypo pigs used in this study

Name	Body weight (g)	Brain weight (g)	Genotype	Age
WT-1	413	20.99	$DUOX2^{+/+}$	P0
WT-2	488	25.81	$DUOX2^{+/+}$	P0
WT-3	359	24.62	$DUOX2^{+/+}$	P0
WT-4	576	22.08	$DUOX2^{+/+}$	P0
WT-5	622	25.42	$DUOX2^{+/+}$	P0
WT-6	496	26.05	$DUOX2^{+/+}$	P0
WT-7	425	30.6	$DUOX2^{+/+}$	P0
WT-8	408	28.22	$DUOX2^{+/+}$	P0
Hypo-1	596	15.04	$DUOX2^{D409G/D409G}$	P0
Hypo-2	605	21.73	$DUOX2^{D409G/D409G}$	P0
Hypo-3	426	17.51	$DUOX2^{D409G/D409G}$	P0
Hypo-4	492	20.49	$DUOX2^{D409G/D409G}$	P0
Hypo-5	560	19.55	$DUOX2^{D409G/D409G}$	P0
Hypo-6	449	22.21	$DUOX2^{D409G/D409G}$	P0

Table S2. Representative GO terms of specifically expressed genes in Astro-1 and Astro-2

Astro	ID	Description	pvalue	p.adjust	qvalue	geneID	Count
Astro-1	GO:0043043	peptide biosynthetic process	1.61E-28	1.03E-25	9.87E-26	ZFP36L2/RPS12/RPL32/RPS8/RPL34/RPS15/RPL10/RPS20/RPL11/QKI/RPLP0/RPS3/RPS29/RPS3A/RPS13/RPL26/RPS16/RPL35/RPL6/RPL27/RPL38/RPL5/RPSA/ZFP36L1/RPS18/RPLP2/RACK1/RPS23/RPS4X/RPS11/RPS21/RPS28/RPS5/RPL29/EEF1B2/EIF4EBP2	36
	GO:0002181	cytoplasmic translation	6.21E-12	1.13E-09	1.09E-09	RPL32/RPLP0/RPS29/RPL26/RPL6/RPSA/RPLP2/RPS23/RPS21/RPS28/RPL29	11
	GO:0042254	ribosome biogenesis	4.82E-09	7.69E-07	7.38E-07	RPS8/RPS15/RPL10/RPLP0/RPL26/RPL7/RPS16/RPL35/RPL6/RPL27/RPSA/RPS21/RPS28	13
	GO:0006417	regulation of translation	1.79E-05	0.001630308	0.001564257	ZFP36L2/RPL10/QKI/RPS3/RPL26/ZFP36L1/RACK1/RPS4X/EIF4EBP2	9
	GO:0008299	isoprenoid biosynthetic process	0.000595686	0.031695476	0.030411354	HMGCS1/FDPS/RBP1	3
Astro-2	GO:0015711	organic anion transport	4.43E-06	0.006483839	0.006037762	SLC4A4/SLC7A10/SLC13A5/SLC1A3/SLC1A2/SLC27A1/LRRC8A/KCNJ10	8
	GO:0045202	synapse	0.000110689	0.01715674	0.013166123	GPR37/GABRB2/KCNK1/ATP2A2/GABRA2/HAPLN1/GABRA1/CHRDL1/CPEB4/GRIA3/KCNJ10/OLFM2/SLITRK2	13
	GO:0031012	extracellular matrix	0.000991231	0.028905659	0.022182271	COL11A2/SPARCL1/F3/TNC/HAPLN1/APOE/TIMP3	7
	GO:0045277	respiratory chain complex IV	0.001305417	0.028905659	0.022182271	COX1/COX3/COX2	3
	GO:0098796	membrane protein complex	0.002684541	0.037957252	0.029128485	GABRB2/ITGAV/GJA1/COX1/KCNK1/COX3/GABRA2/COX2/LRRC8A/ATP6/GABRA1/SEC11C/GRIA3/EMC3	14

Table S3. Sequences of the putative TREs predicted in porcine *COL11A2*

TREs	Sequence	Genomic position*
TRE1	GAGCTAttggGGTTCC	-467 to -452
TRE2	AGGGCGgtccgaGGGTCA	-400 to -383
TRE3	GGGTCAgcgaGGGAGG	-388 to -373
TRE4	AGGTGGagattgAGGGTG	-338 to -321
TRE5	TTGTCAGCCTCA	-73 to -62

*Number of base positions upstream (-) of the transcription start sites.

Table S4. List of antibodies

Antibodies	Source	Identifier
Mouse monoclonal anti-NeuN	Millipore	Cat# MAB377; RRID: AB_2298772
Rabbit polyclonal anti-CUX1	Proteintech	Cat# 11733-1-AP; RRID: AB_2086995
Rabbit polyclonal anti-Reelin	ABclonal	Cat# A17936; RRID: AB_2771981
Mouse monoclonal anti-DCX	Novus	Cat# NBP1-92684SS; RRID: AB_11015624
Goat polyclonal anti-SOX9	R&D Systems	Cat# AF3075; RRID: AB_2194160
Rabbit monoclonal anti-PDGFRα	Cell Signaling Technologies	Cat# 3174S; RRID: AB_2162345
Rat monoclonal anti-Ki67	Thermo Fisher Scientific	Cat# 740008T20UG; RRID: AB_3074182
Goat polyclonal anti-Olig2	R&D Systems	Cat# AF2418; RRID: AB_2157554
Rat monoclonal anti-MBP	Millipore	Cat# MAB386; RRID: AB_94975
Rabbit monoclonal anti-Olig2	Abcam	Cat# ab109186; RRID: AB_10861310
Rabbit monoclonal anti-S100β	Abcam	Cat# ab52642; RRID: AB_882426
Mouse monoclonal anti-S100β	Sino Biological	Cat# 100508-MM03; RRID: AB_3696669
Mouse monoclonal anti-GFAP	Millipore	Cat# MAB360; RRID: AB_11212597
Rabbit polyclonal anti-GFAP	Dako	Cat# Z0334; RRID: AB_10013382
Rat monoclonal anti-CTIP2	Abcam	Cat# ab18465; RRID: AB_2064130
Mouse monoclonal anti-COL11A2	Novus	Cat# NBP2-43728; RRID: AB_2941900
Rabbit polyclonal anti-THRA	Abcam	Cat# ab53729; RRID: AB_882974
Rabbit polyclonal anti-THRB	Abcam	Cat# ab5622; RRID: AB_304991
Mouse monoclonal anti-THRA/THRB	Thermo Fisher Scientific	Cat# MA1-215; RRID: AB_325420
Mouse monoclonal anti-GAPDH	Beyotime	Cat# AG019; RRID: AB_2861160
Mouse monoclonal anti-beta-Actin	Boster Biological Technology	Cat# BM0627; RRID: AB_2814866
Rabbit monoclonal anti-SOX2	Cell Signaling Technologies	Cat# 3728; RRID: AB_2194037
Rabbit polyclonal anti-BLBP	Abcam	Cat# ab32423; RRID: AB_880078
Mouse monoclonal anti-TUJ1	Millipore	Cat# MAB1637; RRID: AB_2210524
Rabbit polyclonal anti-GLAST	Proteintech	Cat# 20785-1-AP; RRID: AB_2878738
Rabbit polyclonal anti-ZBTB20	Proteintech	Cat# 23987-1-AP; RRID: AB_2879389

Table S5. List of primer sequences

Name	Forward primer (5'->3')	Reverse primer (5'->3')	Usage
GAPDH	GCAAAGTGGACATTGTCGCCATCA	AGCTTCCCATTCTCAGCCTTGACT	qRT-PCR
THRA	GGCAAGTCACTCTCTGCCTT	AGGTACGCCTCCTGACTCTT	qRT-PCR
THRB	ACCAGGAGTGGGAGCTCATC	CCACCTTCTGGGGCGTTTAC	qRT-PCR
COL11A2	TCCCTGACGGAGAGTACTGG	CACGTAGGAGAACTGCGTGA	qRT-PCR
GFAP	ATTGTAAATGGAGCCCCGCC	TTGTGCTCCTGCTTGGACTC	qRT-PCR
pcDNA3.1-THRA	ATATATGGATCCATGGAACAGAAGC CAAGC	ATATATGAATTCTTAGACTTCCTGATC CTCAA	Plasmid construction
pcDNA3.1-THRB	ATATATGGATCCATGGGATTTCATGG ATCGG	ATATATGAATTCGTCTAATCCTCAAAC ACTTCC	Plasmid construction
pGL3-TREs	ATATATGCTAGCGCCCTTGCGTCGT TCCC	ATATATAAGCTTAAGCACCAGCGGTAC AAGCA	Plasmid construction
pGL3-mTRE1	GAATCGCCGTTCGAATTAGCGAAAA GAGAATCCTAGGCTTCA	TCGCTAATTCGAACGGCGATTCTCCA GTCTCCTTCGCTTTTG	Plasmid construction
pGL3-mTRE2	GAGTCGGATGTTGGAACGTGCGCG AGGGAGGGTTCCCTTTCGCA	CGCGCACGTTCCAACATCCGACTCT GCTTCTTGCAGGCACTCAG	Plasmid construction
pGL3-mTRE3	CGACTGACCGTGTGCGCGTGTTCC CTTTCGCACCCTGCACCA	AACACGCGCACACGGTCAGTCGGAC CGCCCTCTCTGCTTCTT	Plasmid construction
pGL3-mTRE4	ACCCTGCGAAGCGTGCGCGAAGG GTTCCTCTTCGGTGCCTGTGG	CCCTTCGCGCACGCTTCGCAGGGTG GTGGCGGTGGTGCAGGGTG	Plasmid construction
pGL3-mTRE5	CTCACGACGGACCCTGGGCGTCCA ACGGCTCCGGCGGC	CCCAGGGTCCGTCGTGAGAGGGTGA GCGGGCTTGGACG	Plasmid construction
pSicoR-GFP-shRNA-COL11A2	CCTGGTTTGAAGGGAAATGAA	TTCATTTCCCTTCAAACCAGG	Plasmid construction
pX458-sgRNA-COL11A2	CACCGAGGGTGTCCGACAGCTGGG C	AAACGCCCAGCTGTCGGACACCCTC	Plasmid construction
